# Loss of Acot12 contributes to NAFLD independent of lipolysis of adipose tissue

**DOI:** 10.1038/s12276-021-00648-1

**Published:** 2021-07-20

**Authors:** Sujeong Park, Jinsoo Song, In-Jeoung Baek, Kyu Yun Jang, Chang Yeob Han, Dae Won Jun, Peter K. Kim, Brian Raught, Eun-Jung Jin

**Affiliations:** 1 Department of Biological Sciences, College of Natural Sciences, Wonkwang University, Iksan, Jeonbuk Republic of Korea; 2 Asan Institute for Life Sciences, University of Ulsan College of Medicine, Seoul, Republic of Korea; 3 Department of Pathology, Jeonbuk National University Medical School, Jeonju, Republic of Korea; 4 Research Institute of Clinical Medicine of Jeonbuk National University—Biomedical Research Institute of Jeonbuk National University Hospital and Research Institute for Endocrine Sciences, Jeonju, Republic of Korea; 5 School of Pharmacy, Jeonbuk National University, Jeonju, Jeonbuk Republic of Korea; 6 Department of Internal Medicine, Hanyang University College of Medicine, Seoul, Republic of Korea; 7 Department of Biochemistry, University of Toronto, Toronto, ON Canada; 8 Program of Cell Biology, Hospital for Sick Children, Toronto, ON Canada; 9 Department of Medical Biophysics, University of Toronto, Toronto, ON Canada; 10 Princess Margaret Cancer Center, University Health Network, Toronto, ON Canada

**Keywords:** Gene expression, Disease model

## Abstract

In this study, we hypothesized that deregulation in the maintenance of the pool of coenzyme A (CoA) may play a crucial role in the pathogenesis of nonalcoholic fatty liver disease (NAFLD). Specific deletion of Acot12 (*Acot12*^−/−^), the major acyl-CoA thioesterase, induced the accumulation of acetyl-CoA and resulted in the stimulation of de novo lipogenesis (DNL) and cholesterol biosynthesis in the liver. KEGG pathway analysis suggested PPARα signaling as the most significantly enriched pathway in *Acot12*^−/−^ livers. Surprisingly, the exposure of *Acot12*^−/−^ hepatocytes to fenofibrate significantly increased the accumulation of acetyl-CoA and resulted in the stimulation of cholesterol biosynthesis and DNL. Interaction analysis, including proximity-dependent biotin identification (BioID) analysis, suggested that ACOT12 may directly interact with vacuolar protein sorting-associated protein 33A (VPS33A) and play a role in vesicle-mediated cholesterol trafficking and the process of lysosomal degradation of cholesterol in hepatocytes. In summary, in this study, we found that ACOT12 deficiency is responsible for the pathogenesis of NAFLD through the accumulation of acetyl-CoA and the stimulation of DNL and cholesterol via activation of PPARα and inhibition of cholesterol trafficking.

## Non-alcoholic fatty liver disease: Boosting regulatory enzyme a potential treatment

A deficiency in levels of a key enzyme disturbs lipid metabolism in the liver and triggers non-alcoholic fatty liver disease (NAFLD). NAFLD is a common chronic condition in which fat builds up in the liver, heightening the risk of serious health problems. However, the exact mechanisms behind NAFLD are unclear. In experiments on mice, Eun-Jung Jin at Wongwang University in Iksan, South Korea, and co-workers found that deficiencies of an enzyme called ACOT12 can induce NAFLD regardless of dietary intake. The team generated mice without the *Acot12* gene, which boosted the biosynthesis of lipid and accumulation of cholesterol in liver cells. Further examination showed that ACOT12 may directly interact with a protein that prompts cholesterol trafficking and degradation. Loss of ACOT12 activated a signaling pathway that inhibited this process. Their findings may inform NAFLD therapies.

## Introduction

Nonalcoholic fatty liver disease (NAFLD) is the most common chronic liver disease and occurs when fat accumulates due to factors that are closely associated with obesity and type 2 diabetes mellitus. Nonalcoholic steatohepatitis (NASH) is an extreme form of NAFLD that can eventually lead to cirrhosis and hepatocellular cancer. Even though NAFLD is an important risk factor and the leading cause of liver diseases, there is still no effective pharmaceutical treatment. To identify potential targets for NAFLD for therapeutic treatment, it is necessary to unravel and understand the pathophysiological mechanism of NAFLD.

The liver acts as the main regulator of lipid homeostasis by synthesizing fatty acids, oxidizing lipids, storing excess triglycerides (TGs), or taking up and releasing lipoproteins from/to other interacting organs, such as adipose tissue. The major function of adipose tissue is the storage and release of TGs and free fatty acids (FFAs) through lipogenesis and lipolysis, respectively^1^. Previously, it has been suggested that approximately 60% of hepatic TG comes from FFA pools of adipose tissue, and the rest comes from either de novo lipogenesis (DNL) or imbalanced nutrients by unhealthy diets^2^. Increased FFA flux impairs insulin sensitivity and promotes DNL by upregulating the expression levels of sterol regulatory element-binding protein 1c (SREBP-1c) or PPARγ^3,4^ and stimulates hepatic inflammation through activation of the NF-κB signaling pathway and resident hepatic macrophages known as kupffer cells^5^. Although dysregulation in the interaction between adipose tissue and the liver in NAFLD is well described, the molecular and pathological mechanisms of NAFLD are not fully understood due to the multifaceted nature of NAFLD.

Hepatic cholesterol can be regulated by several alternative metabolic pathways: efflux into the blood in the form of very-low-density lipoprotein or via ATP binding cassette transporter 1 (ABCA1), uptake through bile via ATP binding cassette subfamily G member 5 (ABCG5)/ABCG8, and/or deposition as cholesterol esters or substrates for bile acid synthesis. When the cellular cholesterol level is low, cholesterol synthesis and uptake are induced by HMG-CoA reductase (HMGCR) and low-density lipoprotein receptor (LDLR), respectively. Intracellular cholesterol synthesis could also be regulated by the transcription factor SREBP-2 in the endoplasmic reticulum (ER) membrane. Under low cholesterol conditions, SREBP-2 is processed by proteases and travels into the nucleus to upregulate genes involved in cholesterol syntheses, such as LDLR and HMGCR. On the other hand, at high cholesterol levels, genes involved in cholesterol efflux, such as ABCA1, are increased to reverse cellular cholesterol accumulation.

Recent data suggest that the disturbance of hepatic cholesterol homeostasis and the accumulation of free cholesterol (FC) could cause NAFLD^6^. A significant increase in the level of cholesterol, as well as increased levels of TG and FFAs, has been observed in the liver and plasma of NAFLD patients compared to normal patients^7,8^. Deregulation of hepatic genes related to cholesterol anabolism, catabolism, and efflux, leading to increased hepatic cholesterol levels, has been reported in patients with NAFLD^9^. Moreover, consumption of a high-fat diet with cholesterol led to the development of NAFLD and was accompanied by severe histopathological features, including fibrosis and steatosis^10^. However, the regulatory mechanism and responsible factors contributing to cholesterol accumulation in NAFLD remain to be investigated.

Acetyl-CoA is a key intermediate metabolite that is generated through the breakdown of carbohydrates and fatty acids and resynthesized into TG, glucose, or cholesterol. Acetyl-CoA is regulated by several main key enzymes, such as acetyl-CoA synthetase (ACS), adenosine triphosphate (ATP)-citrate lyase (ACLY), citrate synthase (CS), pyruvate dehydrogenase (PDH), acyl-CoA thioesterase (ACOT), acetyl-CoA carboxylase (ACC), and/or acetyl-CoA acetyltransferase (ACAT). ACOTs that hydrolyze a variety of coenzyme A (CoASH) thioesters, including fatty acyl- and acetyl-CoAs, are known to provide important biological and pathological responses by regulating the intracellular CoA pool^11,12^. Loss of ACOT7 in neurons leads to neurological dysfunction and neurodegeneration due to an increased level of long-chain FFAs^13^, and ACOT11 in brown adipose tissue reduces energy expenditure by hydrolyzing acyl-CoA from lipolysis of LDs in response to adrenergic stimuli^14^. ACOT11-null mice develop resistance to diet-induced obesity and hepatic inflammation steatosis by improving glucose homeostasis^15^. However, to date, the biological and pathological functions of various ACOTs remain poorly understood, and emerging evidence suggests the important role of ACOTs in maintaining metabolic homeostasis and diverse biological responses in the liver.

In this study, we explored the important role of ACOT12, an acyl-CoA thioesterase primarily expressed in the liver, kidney, and small intestine in the pathogenesis of NAFLD. We found that deficiency of ACOT12 resulted in the stimulation of DNL and cholesterol synthesis in the liver through the accumulation of acetyl-CoA and activation of peroxisome proliferator-activated receptor (PPAR) α as well as the inhibition of cholesterol trafficking.

## Materials and methods

### Human and animal study

All human studies included patients with NAFLD diagnosed at Hanyang University Hospital, Seoul, Korea, and fourteen normal patients and 61 NAFLD patient samples were selected for this study. The Institutional Review Board of Hanyang University Hospital approved the human biopsy collections (HY-IRB 2017-03-002-002), and written informed consent was obtained from all patients. All animal studies were performed following approval from the Wonkwang University Animal Care and Use Committee and were in compliance with the institutional guidelines. Eight-week-old male C57BL/6N mice were fed ad libitum a normal chow diet (NCD), a high-fat diet (HFD; 60% kcal from fat; Research Diets Inc.), or a high fat-high-cholesterol diet (HFHCD; 40% kcal from fat with added 1.25% cholesterol (D12108C, Research Diets Inc.) for the indicated times. Genetically obese mice (*ob/ob*) were purchased from Central Lab. Animal Inc. (Seoul, Korea) and fed an HFD ad libitum. In simvastatin administration experiments, 10 mg/kg simvastatin (S6196, Sigma) was injected intraperitoneally daily under HFHCD feeding. In fenofibrate administration experiments, 100 mg/kg fenofibrate (F6020, Sigma) dissolved in corn oil was orally administered daily. All animals fasted for 12 h (overnight) before sacrifice, and blood was collected to isolate serum for biochemical analysis. The tissue was immediately fixed for staining and IHC study, and the rest of the tissue was rapidly kept frozen at −80 °C for subsequent analysis.

### Generation of ACOT12 knockout mice

Acot12 KO mice were generated using the RNA-guided endonuclease (RGEN) method. Acot12 exon 1-specific guide RNA (50 ng/μl) and Cas9 mRNA (50 ng/μl) were injected into the cytoplasm of C57BL/6N mouse zygotes and transferred into C57BL/6N pseudopregnant female mice. To identify Acot12 mutant mice, PCR analysis was performed using genomic DNA from live F0 mice. Finally, we obtained Acot12 mutant mice with the deletion of 14 base pairs. The following primer pair was designed to amplify a 140-bp PCR product from the wild-type allele or 126-bp RGEN-induced mutant allele: forward 5′- AGCCAGGACGATGGAGTCGA-3′, reverse 5′-GGTGTCCATCCACTTGAGCA-3′.

### Histological analysis

Tissues were fixed with 10% (v/v) neutral buffered formalin. One part of each liver was embedded in a paraffin block, and the other part was frozen with Tissue-Tek O.C.T. Compound (Sakura) after cryoprotection with 30% sucrose. Paraffin-embedded livers were sliced into 5-μm sections and stained with hematoxylin and eosin (H&E) for immunostaining in this study. Frozen liver sections were cut into 5-μm sections and stained with Oil red O (Sigma) in 60% isopropanol or 0.25 mg/ml filipin. Paraffin-embedded inguinal white adipose tissue was sliced into 5-μm sections and stained with H&E or CellMask Orange Plasma membrane Stain (Thermo Fisher) with DAPI for nuclear staining. Adipocyte area and adipocyte counting were measured using ImageJ software in 6–8 different fields and are presented in a bar graph. For immunostaining, sections were incubated with the following primary antibodies: ACOT12 (MBS273137, Mybiosource), FASN (GTX109833, GeneTex), SCD1 (ab19862, Abcam), PPARα (ab8934, Abcam), and HA-tag (3724, Cell Signaling). Positive staining was visualized by detection with DAB Peroxidase (HRP) Substrate (SK-4105, Vector Laboratories), and counterstaining with hematoxylin. Histological images were acquired with a light microscope (Thermo Fisher).

### Lentivirus production and injection into the mouse tail vein

To prepare lentiviral particles for injection, 293T cells were transfected with pLenti-GIII-CMV or pLenti-GIII-CMV-*Acot12*-HA using a third-generation packaging system mix (Abm) according to the manufacturer’s protocol. After 2 days, 20 ml of virus-containing medium was collected and concentrated with a Lenti-X concentrator (Takara) according to the manufacturer’s protocol. Mice were then injected six times (in 2 weeks) by tail vein injection with 100 μl of the concentrated virus.

### Isolation and culture of primary hepatocytes and primary adipocytes from the stromal vascular fraction (SVF)

Hepatocytes were isolated using the collagenase perfusion technique from 8-week-old male *Acot12*^+/+^ mice and *Acot12*^−/−^ mice anesthetized by isoflurane. The hepatocytes were cultured in Dulbecco’s modified Eagle’s medium (DMEM) high glucose (4.5 g/L) (Gibco) supplemented with 10% fetal bovine serum (Gibco), 100 U/ml penicillin–streptomycin, and 0.1 μM dexamethasone in a 5% CO_2_ atmosphere at 37 °C. Since the expression level of Acot12 is continuously decreased after plating^16^, all primary hepatocytes were sampled within 2 days after plating. Mouse stromal vascular fraction (SVF) cells were isolated from white adipose tissue of *Acot12*^+/+^ or *Acot12*^−/−^ mice using a previously reported method^17^. Briefly, iWAT was collected from *Acot12*^+/+^ or *Acot12*^−/−^ mice and digested in a collagenase medium. Digested iWAT was centrifuged, and mature adipose cells and SVF cells were separated. Resuspended SVF cells were plated on collagen-coated plates and incubated with 1 μM rosiglitazone in the differentiation medium for adipocyte differentiation.

### Cell culture and fluorescence microscopy

HeLa cells and AML12 cells were cultured in DMEM-high glucose (4.5 g/L) (Gibco) supplemented with 10% fetal bovine serum (Gibco) and 100 U/ml penicillin–streptomycin (pen–strep) in a 5% CO_2_ atmosphere at 37 °C. The NCTC clone 1469 normal mouse liver cell line was cultured in DMEM high glucose supplemented with 10% horse serum and 100 U/ml pen–strep in a 5% CO_2_ atmosphere at 37 °C. Empty vector (pLKO.1) or pLKO-sh*Acot12* packaged lentivirus particles were transfected into AML12 cells for 24 h. Sodium acetate (20 mM) was added 24 h after transfection and then fixed using 4% PFA. Primary hepatocytes isolated from *Acot12*^*+/+*^ and *Acot12*^−/−^ mice have treated with sodium acetate buffer or 50 μM fenofibrate and transfected with empty vector (pcDNA 3.1) or pcDNA-*Ppara* using Lipofectamine 3000 (L3000015, Thermo Fisher). Fixed AML12 cells and primary hepatocytes were incubated using 2 μM BODIPY^493/503^ (D3922, Thermo Fisher) for neutral lipid staining and counterstained with DAPI. For intracellular cholesterol staining of AML12 cells and primary hepatocytes, 0.05 mg/ml filipin in phosphate-buffered saline (PBS) with 10% fetal bovine serum was stained overnight after 1.5 mg/ml glycine incubation. NCTC clone 1469 cells were treated with 50 μg/ml water-soluble cholesterol for 24 h and then fixed in 4% PFA. Fixed NCTC clone 1469 cells were permeabilized with 0.1% Triton X-100 in PBS. Primary anti-ACOT12 antibody (1:100 dilution) and FITC-conjugated rabbit secondary antibody (1:200 dilution) were used for immunocytochemistry. EGFP-tagged VPS33A and TagRFP-tagged ACOT12 expression plasmid DNA were transfected into HeLa cells using Lipofectamine 3000 reagent, and LysoTracker (L12492, Thermo Fisher) and BODIPY cholesterol (24618, Cayman Chemical) was used for focal contact. Images were acquired with a light microscope in an EVOS FL Auto imaging system (Thermo Fisher). Relative fluorescence intensity was measured by ImageJ software to determine ACOT12 expression and the LysoTracker-BODIPY cholesterol merge ratio.

### Glucose tolerance test (GTT) and insulin tolerance test (ITT)

*Acot12*^+/+^ and *Acot12*^−/−^ mice were intraperitoneally injected with glucose (2 g/kg) or insulin (0.75 U/kg) (I-034, Sigma) after overnight fasting (16 h) or 4 h fasting, and blood glucose levels were measured at the indicated time points using a glucometer and glucose test strips.

### Immunoblotting

Twenty milligrams of liver tissue were homogenized with RIPA buffer (Cell Signaling) supplemented with PMSF protease inhibitor (Thermo Fisher) for whole lysates. Fifty micrograms of protein were run on sodium dodecyl sulfate–polyacrylamide gel electrophoresis gels, transferred to nitrocellulose membranes, and then incubated with the following primary antibodies: ACOT12 (MBS273137, Mybiosource), GAPDH (AP0066, Bioworld), and PPARα (ab8934, Abcam). The blotted nitrocellulose membranes were developed using horseradish peroxidase (HRP)-conjugated secondary antibodies (α-Rabbit 1:2000 dilution) purchased from Bethyl Laboratories, and the immunoreactive proteins were detected with SuperSignal West Pico PLUS Chemiluminescent Substrate (Thermo Fisher).

### qRT-PCR

Total RNA was isolated from liver tissue using RNAiso Plus (TaKaRa) according to the manufacturer’s instructions. Then, 1 μg of RNA was reverse transcribed using 5× All-In-One RT Master Mix (with AccuRT Genomic DNA Removal Kit) (G492, Abm). Target mRNA levels were amplified using gene-specific primers (Supplementary Table 1) with Step-One-Plus real-time PCR systems (Applied Biosystems). All PCRs were performed in triplicate. The relative expression level of each gene was normalized to the 18S rRNA expression level.

### RNA-seq analysis

RNA sequencing was performed using an Illumina HiSeq4000 instrument, and libraries were quantified using quantitative real-time PCR (qPCR). RNA library quality was checked using an Agilent Technologies 2100 Bioanalyzer. The raw data were calculated as fragments per kilobase of transcript per million mapped reads of each sample using cufflinks software. Data were transformed logarithmically and normalized using the quantile normalization method.

### Statistical analysis

The results are expressed as the mean ± SEM. The mean values for RNA levels and biochemical data from two experimental groups were compared by unpaired two-tailed Student’s *t* test. When more than two treatment groups were compared, one- or two-way ANOVA for multiple comparisons was used in most studies, as noted in the figure legends. Statistical tests with *P* < 0.05 were considered significant, and significance is denoted as *P < 0.05, **P < 0.01, ***P < 0.001 and ****P < 0.0001. All tests used GraphPad Prism software (GraphPad).

## Results

### ACOT12 is closely related to nonalcoholic fatty liver disease

**Fig. 1:**
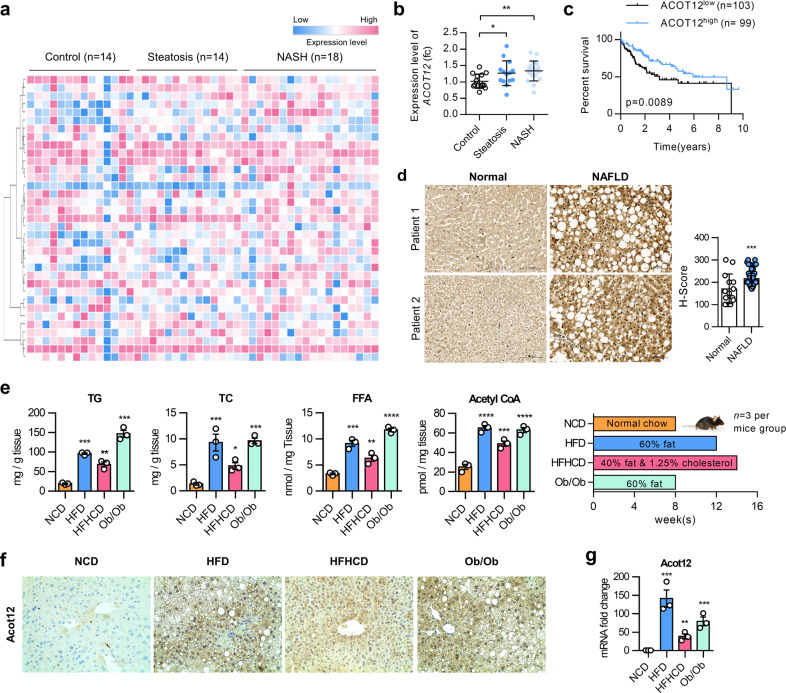
ACOT12 in acetyl-CoA metabolism is involved in NAFLD. **a** Transcription level of 35 enzymes related to acetyl-CoA metabolism in the GSE48452 dataset. **b** Transcription level of *ACOT12* in the GSE48452 dataset. **c** The Kaplan–Meier plot of HCC patient survival with no alcohol consumption in the high-ACOT12 and low-ACOT12 groups. **d** Representative image of immunohistochemical staining of ACOT12 from normal (*n* = 14) and steatotic patient (*n* = 61) liver biopsies. **e** Hepatic triglyceride (TG), total cholesterol (TC), free fatty acid (FFA), and acetyl-CoA levels in NCD, HFD, HFHCD, and ob/ob mice (*n* = 3 mice per group). The diet chow strategy is presented as a bar graph. **f** Representative immunohistochemical staining of ACOT12 from normal chow diet (NCD)-, high-fat diet (HFD)-, and high-fat high-cholesterol diet (HFHCD)-fed wild-type mice and HFD ob/ob mouse livers. Scale bars, 100 μm. **g** Transcription level of *Acot12* in NCD, HFD, HFHCD, and ob/ob mice (*n* = 3 per group). All data are presented as the mean ± SEM. Statistical differences among three or more groups were performed using one-way ANOVA, followed by Dunnett’s multiple comparison test (**f**, **g**) or Fisher’s LSD post hoc test (**b**). *n.s* = nonsignificant; *P < 0.05; **P < 0.01; ***P < 0.001; ****P < 0.0001.

To identify the most relevant and responsible genes of the acetyl-CoA metabolic process (GO: 0006084) underlying the pathogenesis of NAFLD, we performed in silico analysis using Gene Expression Omnibus (GEO, GSE48452)^18^ data of human patient liver biopsy of normal liver, NASH, and steatosis (Fig. 1a). Among key regulators in acetyl-CoA metabolism, the expression level of ACOT12 was the most significantly altered in both steatosis and NASH compared to normal liver tissue, and its expression gradually changed during the progression of NAFLD (Fig. 1b and Supplementary Fig. 1). A previous report suggested that ACOT12 is closely associated with the metastasis of hepatocellular carcinoma (HCC) and survival of HCC patients^19^. Overall survival was significantly reduced in HCC patients with low expression levels of ACOT12. In addition, The Cancer Genome Atlas (TCGA) database of HCC patients showed that a low expression level of ACOT12 reduced the survival rate (Fig. 1c). Here, we found an increased expression level of ACOT12 in fatty liver patients with no alcohol consumption compared to normal patients, suggesting the possible involvement of ACOT12 in NAFLD (Fig. 1d). To further confirm the involvement of ACOT12 in NAFLD, wild-type mice or genetically obese mice (ob/ob) were fed an HFD or HFHCD to induce fatty liver and samples stained with H&E and Oil red O (Supplementary Fig. 2a). Significantly increased levels of hepatic TG, total cholesterol (TC), FFAs, and acetyl-CoA were observed in HFD or HFHCD mice or HFD ob/ob mice compared to NCD mice (Fig. 1e). The expression level of ACOT12 was dramatically increased in the livers of HFD and HFHCD mice compared to the livers of mice fed an NCD (Fig. 1f). Consistent with the analysis of human GEO data, among genes in acetyl-CoA metabolism, ACOT12 was one of the most significantly increased genes in the liver of HFD or HFHCD-Wt mice or HFD ob/ob mice (Fig. 1g and Supplementary Fig. 2b).

### *Acot12*^−/−^ mice have increased lipid and cholesterol synthesis independent of a high-fat diet

**Fig. 2:**
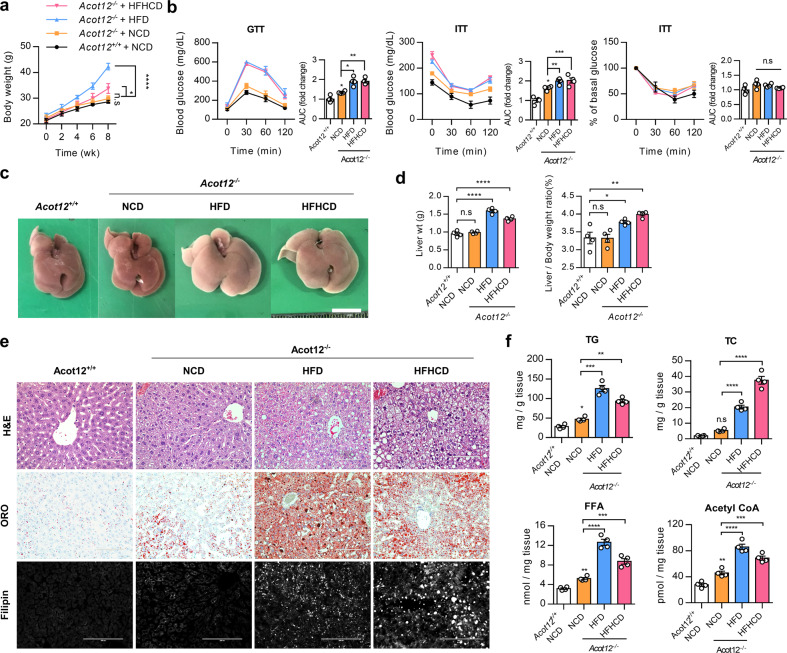
Acot12 deficiency induces severe fat accumulation in the liver under normal chow diet (NCD), high-fat diet (HFD), and high-fat high-cholesterol diet (HFHCD) challenges. Male C57BL/6N wild-type mice (*Acot12*^*+/+*^) and *Acot12*^−/−^ mice were fed an NCD, HFD, or HFHCD for 8 weeks. **a** Body weight of *Acot12*^+/+^ mice fed an NCD or *Acot12*^−/−^ mice fed an NCD, HFD, or HFHCD (*n* = 4). **b** Oral glucose tolerance test (GTT) and insulin tolerance test (ITT). The glucose value in the area under the curve (AUC) was measured for both the GTT and ITT in all *Acot1*2^+/+^ or *Acot12*^−/−^ mice. **c** Photographs of the liver of an NCD-fed *Acot12*^+/+^ mouse and NCD-, HFD-, and HFHCD-fed *Acot12*^−/−^ mouse livers. Scale bars = 1 cm (lower). **d** Liver weight (left) and liver weight to body weight (right). **e** Representative liver staining of hematoxylin and eosin (H&E), Oil Red O (ORO), and filipin. Scale bars, H&E, ORO; 100 μm, filipin; 200 μm. **f** Hepatic levels of triglyceride (TG), total cholesterol (TC), free fatty acid (FFA), and acetyl-CoA in NCD *Acot12*^+/+^ or NCD, HFD, and HFHCD *Ac*ot12^−/−^ liver (*n* = 4). All data are presented as the mean ± SEM. Statistical differences among three or more groups were performed using one-way ANOVA (**b**, **d**, **f**) or two-way ANOVA (**a**), followed by Dunnett’s multiple comparison test. n.s. = nonsignificant; *P < 0.05; **P < 0.01; ***P < 0.001; ****P < 0.0001.

To fully understand the role of Acot12 in the pathogenesis of NAFLD, Acot12 knockout (KO, *Acot12*^−/−^) mice were generated using RGEN-induced mutant alleles in the Acot12 gene, and the efficiency of Acot12 KO was confirmed by immunoblotting and immunostaining (Supplementary Fig. 3). To gain insight into the underlying mechanism in ACOT12 deficiency-induced NAFLD, 8-week-old *Acot12*^+/+^ and *Acot12*^−/−^ mice were challenged with NCD, HFD, and HFHCD for 8 weeks. Interestingly, impaired glucose tolerance and insulin resistance were observed in NCD *Acot12*^−/−^ mice without a significant increase in body weight compared to NCD *Acot12*^*+/+*^ mice, but the severity of impaired glucose tolerance and insulin resistance was significantly greater in HFD or HFHCD *Acot12*^−/−^ mice (Fig. 2a, b, and Supplementary Fig. 4a, b). Increased liver weight and mild hepatomegaly were observed in HFD and HFHCD *Acot12*^−/−^ mice compared to NCD *Acot12*^*+/+*^ mice (Fig. 2c, d). Moreover, NAFLD features, such as increased levels of fat, hepatic TG, TC, FFA, and acetyl-CoA accumulation, were observed in all *Acot12*^−/−^ mouse diet groups fed an HFD, and HFHCD was more pronounced (Fig. 2e, f, Supplementary Fig. 4c, d, Supplementary Fig. 5 and Supplementary Fig. 6). To identify gene groups associated with steatotic livers of HFD *Acot12*^−/−^ mice, we performed gene set enrichment analysis of RNA sequencing data using HFD *Acot12*^*+/+*^ and *Acot12*^−/−^ livers and found dysregulation of lipid homeostasis genes in *Acot12*^−/−^ livers (Supplementary Fig. 7a). The expression levels of genes in the DNL and mevalonate pathways were significantly increased in HFD *Acot12*^−/−^ mice compared to HFD *Acot12*^*+/+*^ mice (Supplementary Fig. 7b, c).

**Fig. 3:**
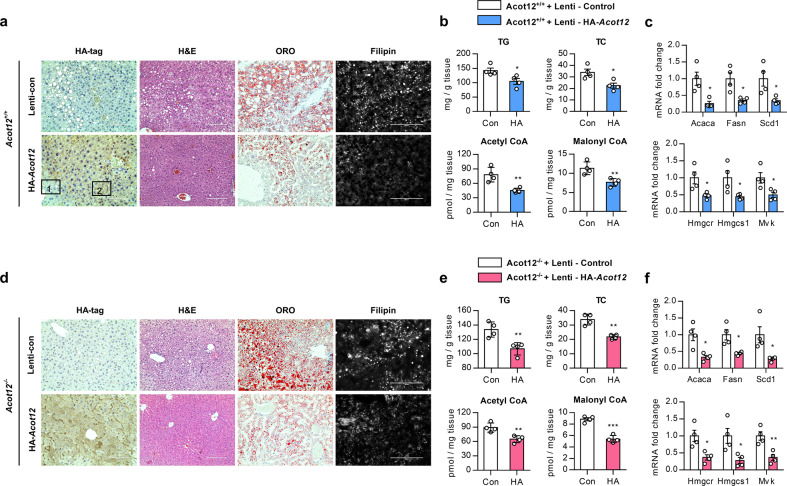
Overexpression and restoration of Acot12 alleviates NAFLD in HFHCD *Acot12*^−/−^ mice. **a** Eight-week-old normal C57BL/6 male mice fed an HFHCD for 14 weeks were injected with lentivirus containing control vector (lenti-con) or HA-tagged Acot12 vector (HA-*Acot12*) after 8 weeks of HFHCD feeding (*n* = 4). Representative liver image of immunostaining of HA-tag, H&E, Sirius Red, Oil Red O, and filipin staining in C57BL/6 mice injected with lentiviruses containing HA-*Acot12* or control vector (lenti-con). A magnified image shows the HA-negative site and HA-positive site in HA-*Acot12*-injected mice. Scale bars, 200 μm. **b** Hepatic TG, TC, acetyl-CoA, and malonyl CoA levels of lenti-con- or HA-*Acot12*-injected 14-week HFHCD *Acot12*^*+/+*^ mice. **c** Expression level of genes related to lipogenesis (upper) and cholesterol metabolism (lower) in lenti-con- or HA-*Acot12*-injected 14-week HFHCD *Acot12*^*+/+*^ mouse livers (*n* = 4). **d** Eight-week-old male *Acot12*^−/−^ mice injected with lentivirus containing lenti-con or HA-*Acot12*. After injection, both mouse groups were fed an HFHCD for 8 weeks (*n* = 4). Representative liver image of immunostaining of HA and H&E, Sirius Red, Oil red O, and filipin staining in HFHCD *Acot12*^−/−^ mice injected with HA-*Acot12* or lenti-con. Scale bars, 200 μm. **e** Hepatic TG, TC, acetyl-CoA, and malonyl-CoA levels of lenti-con- or HA-*Acot12*-injected *Acot12*^−/−^ mice fed an HFHCD for 8 weeks. **f** Expression level of genes related to lipogenesis (upper) and cholesterol metabolism (lower) in lenti-con- or HA-*Acot12*-injected 8-week HFHCD *Acot12*^−/−^ mouse livers (*n* = 4). All data are presented as the mean ± SEM. Statistical differences between two groups were determined using unpaired two-tailed Student’s *t* test (**b**, **e**) or multiple comparison tests followed by the Holm–Sidak method. **c**, **f** *P < 0.05; **P < 0.01; ****P < 0.0001.

Next, we investigated whether the exogenous introduction of *Acot12* could rescue the accumulation of lipids and cholesterol (Supplementary Fig. 8a). Intravenous injection of pLenti-GIII-CMV-*Acot12*-HA (HA-*Acot12*) into HFHCD *Acot12*^*+/+*^ mice significantly decreased the ratio of liver weight to body weight and rescued the impaired glucose and insulin tolerance (Supplementary Fig. 8b, c). Hepatic fat and cholesterol accumulation were dramatically decreased by ACOT12 overexpression (Fig. 3a and Supplementary Fig. 8d). Increased levels of hepatic TG, cholesterol, acetyl-CoA, and malonyl CoA as well as the levels of genes involved in lipogenesis and cholesterol synthesis induced by HFHCD were significantly reduced by the overexpression of ACOT12 (Fig. 3b, c). Moreover, impaired glucose and insulin tolerance in HFHCD *Acot12*^−/−^ mice were rescued by intravenous injection of HA-*Acot12* (Supplementary Fig. 9a–c). Hepatic accumulation of LDs, TGs, cholesterol, acetyl-CoA, and malonyl CoA (Fig. 3d, e) and the expression levels of genes in lipogenesis and cholesterol biosynthesis (Fig. 3f) were also markedly decreased by the restoration of ACOT12 in HFHCD *Acot12*^−/−^ mice. Administration of simvastatin, a widely used cholesterol-lowering drug that competitively inhibits HMGCR, also inhibited the accumulation of LDs and TGs in HFHCD *Acot12*^−/−^ mice (Supplementary Fig. 10).

### Increased acetyl-CoA accumulation in *Acot12*^−/−^ mice leads to upregulation of lipid and cholesterol synthesis

As acetyl-CoA is a precursor to fat biosynthesis, we asked whether the increased level of hepatic acetyl-CoA due to ACOT12 deficiency is directly associated with the increased lipid and cholesterol synthesis observed in *Acot12*^−/−^ mice. To test this hypothesis, we depleted Acot12 in mouse hepatic AML12 cells by treating them with an Acot12-specific short hairpin RNA expression vector (shAcot12) (Supplementary Fig. 11a). Similar to our observations in *Acot12*^−/−^ mice, the depletion of Acot12 expression in AML12 cells increased the cellular levels of acetyl-CoA, TG, and TC and the accumulation of intracellular lipids and cholesterol (Supplementary Fig. 11b–d). The expression of genes involved in lipogenesis and cholesterol synthesis was also significantly increased by Acot12 silencing (Supplementary Fig. 11e).

To evaluate whether excess acetyl-CoA could affect the synthesis of hepatic fatty acids and cholesterol, AML12 cells were treated with acetate. We observed a dose-dependent increase in acetyl-CoA levels upon acetate treatment in AML12 cells (Supplementary Fig. 12a). The expression level of Acot12 also increased in a dose-dependent manner with acetate treatment (Supplementary Fig. 12b). In addition, the cellular levels of TG and TC and the expression levels of genes involved in lipogenesis and cholesterol biosynthesis increased with increasing concentrations of acetyl-CoA (Supplementary Fig. 12c–e). These data suggested that acetyl-CoA accumulation or overload by Acot12 deficiency could stimulate the synthesis of lipids and cholesterol.

### ACOT12 binds with VPS33A and regulates the cholesterol trafficking to the lysosomes process

**Fig. 4:**
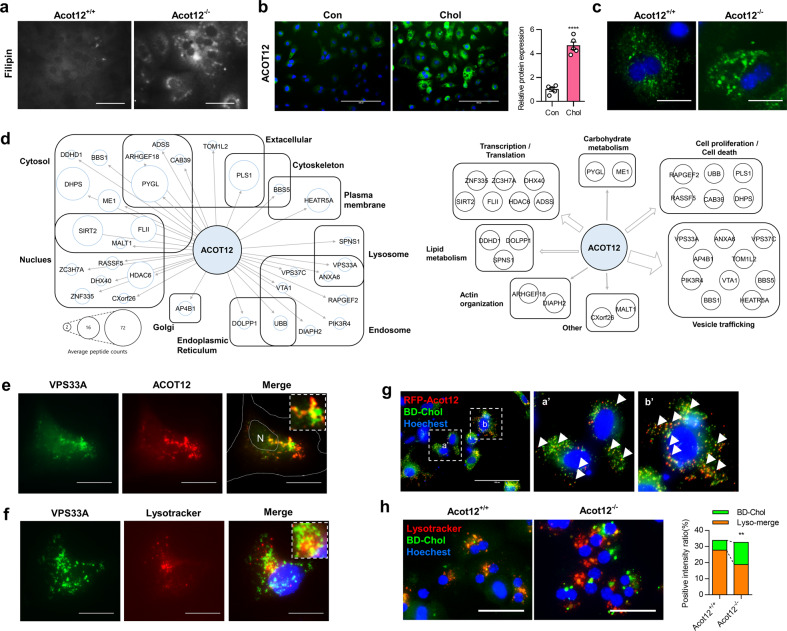
ACOT12 may be involved in cholesterol trafficking by interacting with VPS33A. **a** Representative filipin staining of *Acot12*^*+/+*^ and *Acot12*^−/−^ primary hepatocytes. Scale bars, 25 μm. **b** Representative immunocytochemical staining for ACOT12 in the NCTC clone 1469 normal mouse liver cell line with or without 50 μg/ml water-soluble cholesterol treatment. Scale bars, 100 μm. Fluorescence intensity was measured in five randomly collected images by ImageJ and is visualized as a bar graph (right). **c** Treatment of BODIPY cholesterol in *Acot12*^*+/+*^ and *Acot12*^−/−^ primary hepatocytes. Scale bars, 20 μm. **d** Diagram of the location and function of ACOT12-interacting proteins as assessed by BioID. **e** Merged image of RFP-ACOT12 and EGFP-VPS33A. Scale bars, 20 μm. **f** Representative merged image of EGFP-fused VPS33A (green, EGFP-VPS33A) and LysoTracker staining (red). Scale bars, 20 μm. **g** Merged and magnified images (a’ and b’) of TagRFP-fused ACOT12 (red, RFP-ACOT12) and BODIPY cholesterol (green, BD-Chol). Scale bars, 100 μm. **h** Representative image of LysoTracker and BD-Chol in *Acot12*^*+/+*^ and *Acot12*^−/−^ primary hepatocytes. Nuclei were stained by Hoechst 33342. Scale bars, 50 μm. Fluorescence intensity was measured in ten randomly collected images by ImageJ and is visualized as a bar graph (right). All data are presented as the mean ± SD. Statistical differences between the two groups were determined using an unpaired two-tailed Student’s *t* test. **P < 0.01.

ACOT12, also known as STARD15, consists of esterase domains and a C-terminal steroidogenic acute regulatory-related lipid transfer (START) domain^20^. Fifteen STARD proteins have been found in mammals and are known to play a role in transferring cholesterol and lipids between cellular organelles. STARD1 moves cholesterol to the mitochondrial inner membrane, and STARD3 (MLN64) conveys cholesterol from the ER to endosomes^21,22^. Moreover, STARD4 binds with intracellular FC, and STARD5 and STARD6 contribute to cholesterol trafficking^23–25^. In *Acot12*^−/−^ primary hepatocytes, accumulation of cellular cholesterol as assessed by filipin staining was observed (Fig. 4a), and exposure of primary hepatocytes to water-soluble cholesterol-induced the expression level of ACOT12 (Fig. 4b). Moreover, treatment of *Acot12*^−/−^ primary hepatocytes with BODIPY cholesterol-induced cholesterol accumulation (Fig. 4c), suggesting the possible involvement of ACOT12 in cholesterol trafficking.

To gain greater insight into the function and role of ACOT12 in cholesterol homeostasis, we performed proximity-dependent biotin identification (Bio-ID) analysis to identify possible interacting partners. Analysis of Bio-ID showed that ACOT12 interacted predominately with endosomal and lysosomal proteins (Fig. 4d). One such lysosomal protein is the vesicle trafficking protein vacuolar protein sorting-associated protein 33A (VPS33A), which is known to play a role in vesicle-mediated protein trafficking to lysosomal compartments. To examine whether ACOT12 may interact with VPS33A, we examined its subcellular localization in HeLa cells by confocal fluorescence microscopy and found that ACOT12 colocalized on VPS33A-positive structures (Fig. 4e) and that VPS33A partially colocalized on lysosomes (Fig. 4f). As VPS33A is localized on both lysosomes and endosomes, these images suggest that ACOT12 may be localized on lysosomes.

The homotypic fusion and vacuole protein sorting (HOPS) complex composed of six VPS subunits, including VPS33A, is required for the trafficking of internalized cholesterol by mediating endosome–lysosome fusion. To investigate whether ACOT12 is required for cholesterol trafficking within the endosome–lysosome pathway, we examined TagRFP-fused ACOT12 (RFP-*Acot12*) with BODIPY cholesterol and found that they colocalize with each other (Fig. 4g). Next, we examined whether Acot12 KO cells can disrupt cholesterol trafficking by examining the subcellular localization of BODIPY cholesterol in *Acot12*^−/−^ primary hepatocytes. In wild-type primary hepatocytes, the majority of lysosomes contained cholesterol (Fig. 4h: *Acot12*^*+/+*^). However, the colocalization of BODIPY cholesterol and lysosomes was lost in *Acot12*^−/−^ primary hepatocytes, suggesting that Acot12 may be required for the trafficking of cholesterol through the endosome–lysosome pathway.

### ACOT12 deficiency induces overactivation of PPARα that leads to the accumulation of hepatic acetyl-CoA and cholesterol

**Fig. 5:**
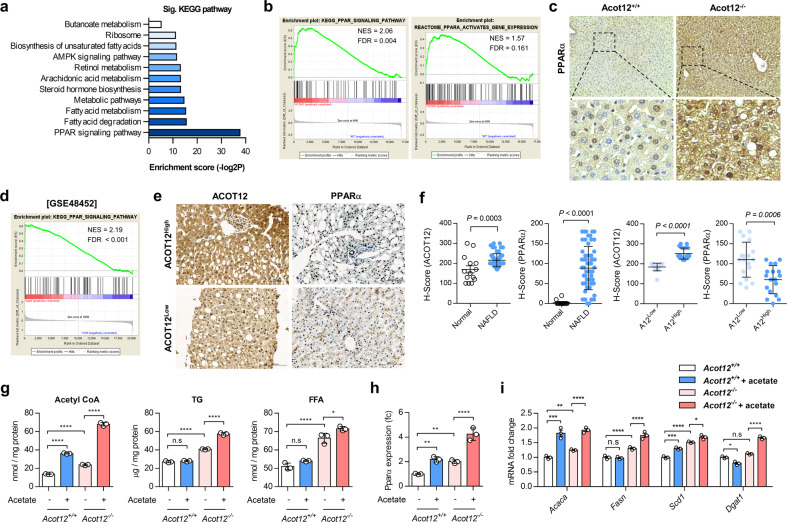
ACOT12 deficiency results in cholesterol accumulation via stimulation of PPARα. **a** KEGG pathway analysis of RNA sequencing data of HFD *Acot12*^−/−^ livers compared with *Acot12*^*+/+*^ livers. **b** Gene set analysis of RNA sequencing data of HFD *Acot12*^−/−^ livers. **c** Immunostaining of PPARα in 8-week HFD-fed *Acot12*^*+/+*^ and *Acot12*^−/−^ livers. The magnified image shows representative PPARα-positive sites in HFD-fed *Acot12*^*+/+*^ and *Acot12*^−/−^ mouse livers. Scale bars, 200 μm. **d** Gene set analysis of human liver biopsy of a normal, NASH, steatosis patient-related GEO dataset (GSE48452). **e** Representative image of immunohistochemical staining of PPARα in liver biopsies from patients with steatosis with high ACOT12 expression (*n* = 18) and patients with steatosis with low ACOT12 expression (*n* = 18). **f** Histological score of immunohistochemical staining of ACOT12 and PPARα in liver biopsies of steatosis patients. High and low expression of ACOT12 sorted by *H*-score of 200. **g** Levels of TG and TC in *Acot12*^*+/+*^ and *Acot12*^−/−^ mouse primary hepatocytes with or without acetate. **h** Transcription level of *Ppara* in *Acot12*^*+/+*^ and *Acot12*^−/−^ mouse primary hepatocytes with or without acetate. **i** Expression level of lipogenesis genes in *Acot12*^*+/+*^ and *Acot12*^−/−^ mouse primary hepatocytes with or without acetate. All data are presented as the mean ± SEM. Statistical differences between two groups were determined using unpaired two-tailed Student’s *t* test (**f**); statistical differences among four groups were performed using one-way ANOVA, followed by the Holm–Sidak multiple comparison test (**g**, **h**); statistical differences in individual gene expression among four groups were performed using two-way ANOVA, followed by Tukey’s multiple comparison test (**i**). n.s. nonsignificant; *P < 0.05; **P < 0.01; ***P < 0.001; ****P < 0.0001.

To identify a key signaling pathway responsible for the accumulation of lipids and cholesterol in *Acot12*^−/−^ livers, RNA sequencing was performed, and the enrichment of biological pathways was assessed by KEGG (Kyoto Encyclopedia of Genes and Genomes) analysis. KEGG pathway analysis suggested PPARα as the most significantly enriched signaling pathway in HFD *Acot12*^−/−^ livers (Fig. 5a, b). Introduction of shAcot12 into murine alpha mouse liver 12 (AML12) cells significantly increased the expression level of *Ppara* (Supplementary Fig. 13a). Furthermore, the expression level of PPARα was also significantly increased in HFD *Acot12*^−/−^ livers (Fig. 5c and Supplementary Fig. 13b). In silico analysis using GSE48452 indicated increased levels of PPARα in NASH patients (Fig. 5d). Consistent with this, we also found an increased level of PPARα in HFD *Acot12*^*+/+*^ livers (Supplementary Fig. 13c). Consistent with the *Acot12*^−/−^ mouse liver data, PPARα protein levels were inversely proportional to the protein levels of ACOT12 in the fatty liver patient samples (Fig. 5e, f).

Several studies suggested that newly synthesized fatty acids and other lipid metabolites could act as ligands for PPARα. Long-chain fatty acids generated de novo by fatty acid synthase (FASN) could serve as ligands for PPARα in the liver^26^. High malonyl-CoA induces the accumulation of long-chain fatty acyl-CoAs by inhibiting carnitine palmitoyltransferase-1 (CPT-1) and results in the activation of PPARα in the mouse heart^27^. Several saturated fatty acids, such as palmitate (16:0), myristate (14:0), and stearate (18:0), also bind to PPARα and act as ligands for PPARα, similar to other unsaturated fatty acids^28^. To investigate whether high acetyl-CoA induced by ACOT12 deficiency could induce the activation of PPARα, primary hepatocytes isolated from *Acot12*^*+/+*^ and *Acot12*^−/−^ livers were exposed to acetate for acetyl-CoA overload. With acetate treatment, the cellular levels of acetyl-CoA, TGs, and FFAs (Fig. 5g) and the expression levels of PPARα (Fig. 5h) and lipogenic genes (Fig. 5i) were significantly increased in *Acot12*^−/−^ primary hepatocytes.

Previous studies have suggested that the activation of PPARα could improve hepatic steatosis, inflammation, and fibrosis by enhancing fatty acid oxidation^29,30^. However, in ACOT12 deficiency, an increased expression level of PPARα worsens the symptoms of NAFLD through the accumulation of lipids and cholesterol possibly converted from acetyl-CoA. To confirm the vicious action of PPARα under ACOT12 deficiency in NAFLD, primary hepatocytes isolated from *Acot12*^*+/+*^ and *Acot12*^−/−^ livers were transfected with lentiviral vectors containing *Ppara* (Supplementary Fig. 14a). The cellular levels of acetyl-CoA, FFAs, cholesterol (Supplementary Fig. 14b–e), DNL, and cholesterol synthesis (Supplementary Fig. 14f, g) were significantly increased in *Acot12*^−/−^ primary hepatocytes but not in *Acot12*^*+/+*^ primary hepatocytes. These data suggested that increased acetyl-CoA by ACOT12 deficiency produces lipids that activate PPARα, which stimulates DNL.

**Fig. 6:**
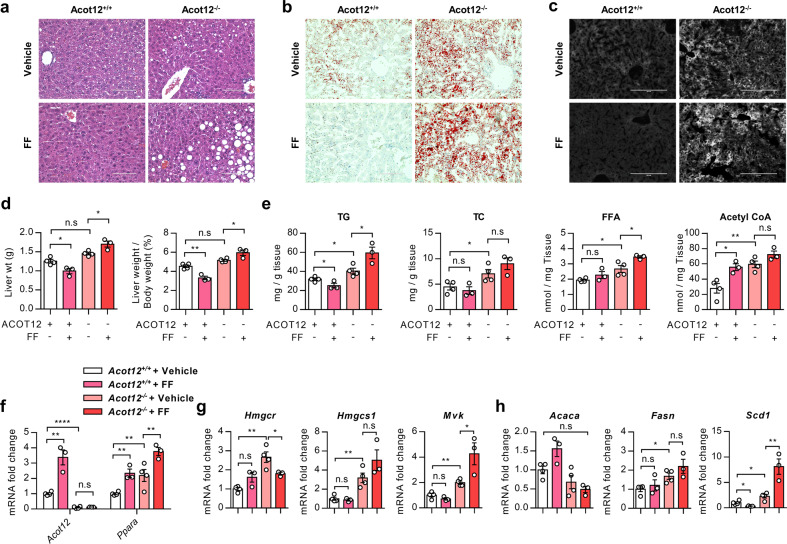
Fenofibrate did not reduce NAFLD features in *Acot12*^−/−^ mice. **a** Representative H&E staining of *Acot12*^*+/+*^ and *Acot12*^−/−^ mouse livers administered vehicle (corn oil) or fenofibrate (FF, 100 mg/kg). *n* = 3–4 per mice group. Scale bars, 100 μm. **b** Representative ORO staining in *Acot12*^*+/+*^ and *Acot12*^−/−^ mouse livers administered vehicle or FF. Scale bars, 100 μm. **c** Representative filipin staining in *Acot12*^*+/+*^ and *Acot12*^−/−^ mouse livers administered vehicle or FF. Scale bars, 100 μm. **d** Liver weight and the ratio of liver weight to body weight in vehicle- or FF-administered HFHCD *Acot12*^+/+^ or *Acot12*^−/−^ mice. **e** Hepatic TG, TC, FFA, and acetyl-CoA levels in *Acot12*^*+/+*^ and *Acot12*^−/−^ mouse livers administered vehicle or FF. **f** Expression levels of *Acot12* and *Ppara* in *Acot12*^*+/+*^ and *Acot12*^−/−^ mouse livers administered vehicle or FF. **g** Expression levels of cholesterol biosynthesis genes in *Acot12*^*+/+*^ and *Acot12*^−/−^ mouse livers administered vehicle or FF (*n* = 3–4 mice per group). **h** Expression levels of lipogenic genes in *Acot12*^*+/+*^ and *Acot12*^−/−^ mouse livers administered vehicle or FF (*n* = 3–4 mice per group). All data are presented as the mean ± SEM. Statistical differences among four groups were performed using one-way ANOVA (**d**, **e**, **g**, **h**) followed by Tukey’s multiple comparison test or multiple *t* test, followed by Holm–Sidak’s multiple comparison test (**f**). n.s. nonsignificant; *P < 0.05; **P < 0.01; ***P < 0.001; ****P < 0.0001.

To further test whether PPARα was upregulated in *Acot12*^−/−^ hepatocytes to counter the cellular increase in lipids, we treated the cells with fenofibrate, a known PPARα agonist. As expected in *Acot12*^*+/+*^ hepatocytes, fenofibrate treatment increased PPARα expression and acetyl-CoA levels. However, we found that the activation of PPARα by fenofibrate failed to reduce FFAs or TC but instead resulted in a greater increase in cellular lipids and cholesterol in the *Acot12*^−/−^ cells (Supplementary Fig. 15a–e). Moreover, the expression levels of genes involved in DNL and cholesterol synthesis were also significantly increased upon exposure to fenofibrate in *Acot12*^−/−^ primary hepatocytes but not in wild-type primary hepatocytes (Supplementary Fig. 15f, g). To validate our findings in cultured primary hepatocytes, we treated *Acot12*^−/−^ mice on an HFHCD with fenofibrate for 6 weeks (100 mg/kg/day). Similar to the cultured cells, the fenofibrate-treated HFHCD *Acot12*^*+/+*^ mice showed evidence of decreased accumulation of LDs and cholesterol and a significant decrease in liver weight, hepatic TGs, and acetyl-CoA compared to untreated animals. On the other hand, in fenofibrate-administered HFHCD *Acot12*^−/−^ mice, the accumulation of hepatic LDs and cholesterol was distinctly increased even though PPARα was successfully increased by fenofibrate administration (Fig. 6a–e). Unlike *Acot12*^*+/+*^ mice, fenofibrate administration to *Acot12*^−/−^ mice induced the expression level of DNL and cholesterol synthesis genes with the activation of PPARα (Fig. 6f, g and Supplementary Fig. 16).

### ACOT12 deficiency induces NAFLD independently from lipolysis of adipose tissue

To determine the time of pathological onset of fatty liver by ACOT12 deficiency, we analyzed adipose and liver tissue in HFD *Acot12*^+/+^ and *Acot12*^−/−^ mice at 2, 4, 6, and 8 weeks. In HFD *Acot12*^−/−^ mice, the accumulation of hepatic fat (Supplementary Fig. 17a) and increased levels of hepatic TG and TC (Supplementary Fig. 17b) were observed after 4 weeks of HFD feeding, and adipose tissue hypertrophy was not significantly changed (Supplementary Fig. 17c, d).

Adipose tissue plays an important role in energy metabolism and contributes to the development of NAFLD. However, the pathological onset of fatty liver in *Acot12*^−/−^ mice suggests the possibility that hepatic fat accumulation may not be influenced by an increase in adipose tissue. To investigate whether fatty liver induced by ACOT12 deficiency occurred independent of adipose tissue, primary hepatocytes isolated from *Acot12*^*+/+*^ and *Acot12*^−/−^ mice were co-cultured with primary adipocytes isolated from adipose-derived SVF (adSVF) cells from the inguinal white adipose tissue (iWAT) of either *Acot12*^*+/+*^ or *Acot12*^−/−^ mice. *Acot12*^*−/−*^ primary hepatocytes showed significantly more lipid droplet (LD) accumulation than *Acot12*^*+/+*^ cells regardless of whether primary adipocytes were cocultured (Supplementary Fig. 18a). In fact, no difference was observed between *Acot12*^−/−^ hepatocytes cocultured with *Acot12*^*+/+*^ and *Acot12*^−/−^ primary adipocytes. Moreover, the expression level of lipogenic genes was also significantly increased in *Acot12*^−/−^ primary hepatocytes cocultured with *Acot12*^*+/+*^ primary adipocytes without alteration of the expression level of lipid transport genes (Supplementary Fig. 18b, c).

## Discussion

As a central regulator of lipid homeostasis, the liver is responsible for the biosynthesis of fatty acids and exports them to other tissues as energy substrates. The disruption of hepatic lipid homeostasis by an imbalance between lipid acquisition and lipid disposal due to deregulation of circulating lipid uptake, DNL, FAO, and/or lipid export may cause the retention of fat and result in the development of NAFLD. It is well-known that FAs released from adipose tissues are closely associated with fatty liver disease^31^. Lipolysis in adipose tissue, i.e., the hydrolysis of TGs by adipose TG lipase (*ATGL*) and monoacylglycerol lipase (*MGL*) results in the release of FAs into the circulation and leads to fat accumulation in the liver^32^. DNL is an integrated metabolic pathway that includes glycolysis, the biosynthesis of saturated fatty acids followed by desaturation, and the formation of TG^33^. Hepatic DNL starts with the conversion of acetyl-CoA into malonyl-CoA by acetyl-CoA carboxylase (ACC) and uses malonyl-CoA as a substrate to produce 16-carbon palmitic acyl-CoA by FASN, which is responsible for the building of new fatty acids. Pharmacologic inhibition or genetic inactivation of hepatic ACC has been predicted to be an attractive approach to the reduction of NAFLD. Mice with targeted mutations that activate ACC constitutively demonstrated histological and clinical signs of NAFLD, whereas liver-directed ACC inhibition reduced hepatic steatosis by suppressing hepatic DNL^34^. Despite the close association between DNL and NAFLD, surprisingly little is known about the sources of acetyl-CoA, fuel in this process.

In normal liver, acetyl-CoA is oxidized in the tricarboxylic acid cycle to form CO_2_ or is condensed in the ketogenic pathway to form acetoacetate or β-hydroxybutyrate^35,36^. NAFLD patients showed dramatic differences in the metabolic fate of acetyl-CoA and elevated rates of pyruvate carboxylase flux, gluconeogenesis, EGP, and oxygen consumption^37–39^. Here, we demonstrated that the development of NAFLD in *Acot12*^−/−^ mice closely reflected the histopathology of human NAFLD by increased DNL and cholesterol biosynthesis through the accumulation of acetyl-CoA. Moreover, coculture of *Acot12*^−/−^ hepatocytes and *Acot12*^*+/+*^ adipocytes showed increased DNL in *Acot12*^−/−^ hepatocytes, and inhibition of lipolysis did not affect hepatic FFAs, TGs, TC, or DNL in *Acot12*^−/−^ livers. These data suggested that fatty liver in *Acot12*^−/−^ mice might result from increased hepatic DNL but not from lipolysis of adipocytes.

Two well-known transcription factors, PPARs, members of the nuclear hormone receptor superfamily and sterol-regulatory element-binding proteins (SREBPs), a subgroup of basic-helix–loop–helix–leucine zipper (bHLH-LZ) transcription factors, play a crucial role in lipid homeostasis, such as fatty acid synthesis and cholesterol metabolism^40^. In particular, PPARα is highly expressed in tissues with high FAO rates, such as liver, heart, skeletal muscle, and brown adipose tissue, and regulates peroxisomal and mitochondrial fatty acid catabolism. PPARα deficiency results in severe steatosis and hepatitis, whereas treatment with the PPARα agonist Wy-14,643 protects against steatosis and steatohepatitis in methionine- and choline-deficient diet-fed mice^29,41^. A large variety of long-chain fatty acids, eicosanoids, and synthetic compounds have been shown to serve as endogenous PPARα ligands and activators^42,43^. Fibrates have been defined as exogenous PPARα ligands, which are widely used to reduce hepatic fat in animal experiments^44^. However, recent clinical and preclinical studies with PPARα agonists such as fenofibrate and clofibrate suggested no significant improvement in NAFLD activity^45^ but rather showed that PPARα activation increases hepatic DNL and cholesterol accumulation^46,47^. In this study, we suggested that accumulated acetyl-CoA could act as an endogenous ligand for PPARα and activate the anabolic synthesis of cholesterol and DNL. Moreover, our BioID analysis suggested the possible interaction between ACOT12 and VPS33A, a core component of the class C core vacuole/endosome tethering complex and the HOPS complex known to play a role in vesicle-mediated protein trafficking to lysosomal compartments. The HOPS complex plays a role in the transport and processing of cholesterol^48,49^. This finding suggested the involvement of ACOT12 in the trafficking of cholesterol by interacting with VPS33A and could serve as a new model of intracellular cholesterol trafficking.

In summary, in this study, we found that ACOT12 deficiency may be responsible for the pathogenesis of NAFLD through the accumulation of cholesterol in the liver and suggest that Acot12 could be a promising therapeutic therapy for controlling NAFLD.

## Supplementary information

supplementary figures and Table

## Data Availability

The datasets generated during and/or analyzed during the current study are available from the corresponding author on reasonable request.
