# Supplementary material for: Loss of Acot12 contributes to NAFLD independent of lipolysis of adipose tissue
Source: Exp Mol Med. 2021 Jul 20;53(7):1159–69. doi: 10.1038/s12276-021-00648-1 (PMC8333268; doi:10.1038/s12276-021-00648-1)
Supplement: Supplementary file 1 — supplementary figures and Table [file 12276_2021_648_MOESM1_ESM.pdf]

**Supplementary figure legends**

**Supplementary Figure 1.** The expression level of genes in acetyl CoA metabolism. The expression level of *ACLY*, *ACSS1*, *ACSS2*, *PDHA1*, *CS*, *ACACA*, and *ACAT1* using microarray data of human NAFLD liver biopsy (GSE48452) ($n$ = 14 (control and steatosis) or 18 (NASH)). Statistical differences among three more groups were performed using one-way ANOVA, followed by Fisher's LSD post-hoc test. *n.s* = non-significant; *$P < 0.05$.

**Supplementary Figure 2.** Feature of NCD, HFD, HFHCD fed wild type mice and HFD Ob/Ob mice. **a** Representative histological image of normal chow diet (NCD), high-fat diet (HFD) and high-fat high-cholesterol diet (HFHCD) fed wild type mice and HFD Ob/Ob mice. Scale bars, H&E and Sirius red, 200μm; Oil red O and Acot12, 100 μm. **b** Transcription level of 7 key enzyme of acetyl CoA metabolism in NCD, HFD, HFHCD and Ob/Ob mice ($n$ = 3 per group). Statistical differences among three more groups were performed using one-way ANOVA, followed by Dunnett's multiple comparison test. *n.s* = non-significant; *$P < 0.05$.

**Supplementary Figure 3.** Generation of Acot12 knockout mice. The colored sequence indicates TALEN-binding sites in Acot12 exon1 (upper). **a** DNA sequences of the Acot12 locus from selected F0 mice were identified by PCR analysis. "-" denotes a deleted nucleotide (lower). **b** Immunoblotting of ACOT12 in *Acot12*$^{+/+}$ or *Acot12*$^{-/-}$ liver. **c** Immunostaining of ACOT12 in *Acot12*$^{+/+}$ or *Acot12*$^{-/-}$ liver.

**Supplementary Figure 4.** Feature of NCD fed *Acot12*$^{+/+}$ and *Acot12*$^{-/-}$ mice. **a** Body weight of

8 weeks NCD fed *Acot12*$^{+/+}$ and *Acot12*$^{-/-}$ mice. **b** GTT and ITT in NCD *Acot12*$^{+/+}$ and *Acot12*$^{-/-}$ mice. Glucose value in the area under the curve (AUC) was measured both GTT and ITT (*n* = 4 mice per group). Percent of basal glucose level about ITT test was presented in right side of ITT test. **c** Representative images of hematoxylin and eosin (H&E), Oil Red O (ORO) and filipin staining in NCD fed *Acot12*$^{+/+}$ and *Acot12*$^{-/-}$ mice liver. Scale bars, H&E, ORO: 100 μm, filipin: 200 μm. **d** The levels of hepatic triglyceride (TG), total cholesterol (TC), free fatty acid (FFA) and acetyl CoA in NCD fed *Acot12*$^{+/+}$ and *Acot12*$^{-/-}$ mice liver (*n* = 4 mice per group). All data are represented as mean ± SEM. Statistical differences among three groups were performed using one-way ANOVA (**b**, **d**) or two-way ANOVA (**a**), followed by Dunnett's multiple comparison test. *, P < 0.05; **, P < 0.01; ***, P < 0.001.

**Supplementary Figure 5.** Feature of HFD fed *Acot12*$^{+/+}$ and *Acot12*$^{-/-}$ mice. **a** Body weight of 8 weeks HFD fed *Acot12*$^{+/+}$ and *Acot12*$^{-/-}$ mice. **b** GTT and ITT in HFD *Acot12*$^{+/+}$ and *Acot12*$^{-/-}$ mice. Glucose value in the area under the curve (AUC) was measured both GTT and ITT (*n* = 4 mice per group). Percent of basal glucose level about ITT test was presented in right side of ITT test. **c** Representative images of hematoxylin and eosin (H&E), Oil Red O (ORO) and filipin staining in HFD fed *Acot12*$^{+/+}$ and *Acot12*$^{-/-}$ mice liver. Scale bars, H&E, ORO: 100 μm, filipin: 200 μm. **d** The levels of hepatic triglyceride (TG), total cholesterol (TC), free fatty acid (FFA) and acetyl CoA in HFD fed *Acot12*$^{+/+}$ and *Acot12*$^{-/-}$ mice liver (*n* = 4 mice per group). All data are represented as mean ± SEM. Statistical differences among three groups were performed using one-way ANOVA (**b**, **d**) or two-way ANOVA (**a**), followed by Dunnett's multiple comparison test. *, P < 0.05; **, P < 0.01; ***, P < 0.001.

**Supplementary Figure 6.** Feature of HFHCD fed *Acot12*$^{+/+}$ and *Acot12*$^{-/-}$ mice. **a** Body weight of 8 weeks HFHCD fed *Acot12*$^{+/+}$ and *Acot12*$^{-/-}$ mice. **b** GTT and ITT in HFHCD *Acot12*$^{+/+}$ and *Acot12*$^{-/-}$ mice. Glucose value in the area under the curve (AUC) was measured both GTT and ITT (*n* = 4 mice per group). Percent of basal glucose level about ITT test was presented in right side of ITT test. **c** Representative images of hematoxylin and eosin (H&E), Oil Red O (ORO) and filipin staining in HFHCD fed *Acot12*$^{+/+}$ and *Acot12*$^{-/-}$ mice liver. Scale bars, H&E, ORO: 100 μm, filipin: 200 μm. **d** The levels of hepatic triglyceride (TG), total cholesterol (TC), free fatty acid (FFA) and acetyl CoA in HFHCD fed *Acot12*$^{+/+}$ and *Acot12*$^{-/-}$ mice liver (*n* = 4 mice per group). All data are represented as mean ± SEM. Statistical differences among three groups were performed using one-way ANOVA (**b**, **d**) or two-way ANOVA (**a)**, followed by Dunnett's multiple comparison test. *, P < 0.05; **, P < 0.01; ***, P < 0.001.

**Supplementary Figure 7.** Gene expression analysis of HFD *Acot12*$^{+/+}$ and *Acot12*$^{-/-}$ mice. **a** Gene set analysis of HFD *Acot12*$^{+/+}$ and *Acot12*$^{-/-}$ mice liver. **b** Transcription level of genes encoding lipogenic enzymes in HFD *Acot12*$^{+/+}$ and *Acot12*$^{-/-}$ liver (*n* = 4). **c** Transcription level of genes encoding cholesterol biosynthesis enzymes in HFD *Acot12*$^{+/+}$ and *Acot12*$^{-/-}$ liver (*n* = 4). All data are represented as mean ± SEM. Statistical differences between two groups were determined using unpaired two-tailed Student's *t* test. *n.s* = non-significant; *$P < 0.05$; **$P < 0.01$; ***$P < 0.001$.

**Supplementary Figure 8.** Overexpression of ACOT12 in *Acot12*$^{+/+}$ mice. **a** Schematic diagram of ACOT12 overexpression (HA-*Acot12*) in *Acot12*$^{+/+}$ mice. **b** The ratio of liver weight to body in HFHCD *Acot12*$^{+/+}$ mice with ACOT12 overexpression. **c** GTT and ITT in

HFHCD *Acot12*$^{+/+}$ mice with ACOT12 overexpression. **d** Magnified image of immunohistochemical staining of HA-tag (Fig. 3A) in HA-Acot12 injected *Acot12*$^{+/+}$ mice liver. All data are represented as mean ± SEM. Statistical differences between two groups were determined using unpaired two-tailed Student's *t* test. *$P < 0.05$; *$P < 0.01$.

**Supplementary Figure 9.** Restoration of ACOT12 in *Acot12*$^{-/-}$ mice. **a** Schematic diagram of ACOT12 restoration (HA-*Acot12*) in *Acot12*$^{-/-}$ mice. **b** The ratio of liver weight to body in HFHCD *Acot12*$^{-/-}$ mice with ACOT12 restoration. **c** GTT and ITT in restoration HFHCD *Acot12*$^{-/-}$ mice with ACOT12 restoration. All data are represented as mean ± SEM. Statistical differences between two groups were determined using unpaired two-tailed Student's *t* test. *n.s* = non-significant; *$P < 0.05$.

**Supplementary Figure 10.** Inhibition of cholesterol synthesis alleviates hepatic fat and cholesterol accumulation in *Acot12*$^{-/-}$ mice. **a** Representative images of H&E, ORO, and filipin staining in vehicle (PBS) or simvastatin (Simva) injected HFHCD *Acot12*$^{-/-}$ livers. 10 mg/kg simvastatin was intraperitoneally injected every day for 8 weeks ($n$ = 5). Scale bars, 200 μm. **b** Hepatic TG and TC level in vehicle or simva injected HFHCD *Acot12*$^{-/-}$ mice ($n$ = 4). All data are represented as mean ± SEM. Statistical differences between two groups were determined using unpaired two-tailed Student's *t* test. **$P < 0.01$.

**Supplementary Figure 11.** The accumulation of acetyl CoA by ACOT12 deficiency is responsible for the stimulation of lipid and cholesterol synthesis. **a** Knockdown efficiency of ACOT12 (sh Acot12) was confirmed by qRT-PCR. **b** Cellular acetyl CoA level of control or

sh Acot12 transfected AML12, mouse normal liver cell line. **c** The level of cellular TG and TC in control or sh Acot12 transfected AML12 cells. **d** Representative image of BODIPY$^{493/503}$ and filipin staining in ACOT12 knockdowned AML12 cells. Scale bars, 50 μm. **e** Expression level of genes involved in lipid and cholesterol biosynthesis in AML12 cells. All data are represented as mean ± SEM. Statistical differences between two groups were determined using unpaired two-tailed Student's *t* test. $^*P < 0.05$; $^{**}P < 0.01$; $^{***}P < 0.001$; $^{****}P < 0.0001$.

**Supplementary Figure 12.** Acetyl CoA overload activates de novo lipogenesis and cholesterol synthesis. **a** Cellular acetyl CoA level of AML12 cells treated with 0, 5, 10, 25 mM acetate for 24h, respectively. **b** Expression of ACOT12 mRNA level in acetate treated AML12 cells. **c** Cellular level of TG and TC in acetate treated AML12 cells. **d** Expression level of genes associated with DNL pathway in acetate treated AML12 cells. **e** Expression level of genes associated with cholesterol synthesis in AML12 cells. All data are represented as mean ± SEM. Statistical differences among four groups were performed using one-way ANOVA, followed by Dunnett's multiple comparison test.

**Supplementary Figure 13.** ACOT12 deficiency stimulates PPARα expression. **a** Expression level of *Ppara* in control or sh Acot12 transfected AML12. **b** Expression level of *Ppara* in HFD $Acot12^{+/+}$ and $Acot12^{-/-}$ liver (left) and immunoblotting of PPARα in HFD $Acot12^{+/+}$ and $Acot12^{-/-}$ liver (right) ($n$ = 4). GAPDH was used as a loading control. **c** Immunostaining of PPARα in HFD $Acot12^{+/+}$ at 0, 2, 4, 8, and 16 weeks. Scale bars, 100 μm. All data are represented as mean ± SEM. Statistical differences between two groups were determined using unpaired two-tailed Student's *t* test **(b)**. $^{**}P < 0.01$; $^{***}P < 0.001$.

**Supplementary Figure 14.** Effect of PPARα overexpression in *Acot12*$^{+/+}$ or *Acot12*$^{-/-}$ primary hepatocyte. **a** Expression level of *Ppara* in empty vector or pcDNA-mPpara (Ppara) transfected *Acot12*$^{+/+}$ and *Acot12*$^{-/-}$ primary hepatocyte. Transfection efficiency confirmed by qRT-PCR. **b** Cellular acetyl CoA level in empty vector or pcDNA-*Ppara* (Ppara) transfected *Acot12*$^{+/+}$ and *Acot12*$^{-/-}$ primary hepatocyte. **c** Cellular FFA (left) and TC (right) level in empty vector or pcDNA-*Ppara* transfected *Acot12*$^{+/+}$ and *Acot12*$^{-/-}$ primary hepatocyte. **d** Representative image of BODIPY 493/503 staining in empty vector or pcDNA-*Ppara* transfected *Acot12*$^{+/+}$ and *Acot12*$^{-/-}$ primary hepatocyte. **e** Representative image of filipin staining in empty vector or pcDNA-*Ppara* transfected *Acot12*$^{+/+}$ and *Acot12*$^{-/-}$ primary hepatocyte. Scale bars, 100 μm. **f** Expression level of genes associated with DNL pathway in empty vector or pcDNA-*Ppara* transfected *Acot12*$^{+/+}$ and *Acot12*$^{-/-}$ primary hepatocyte. **g** Expression level of genes associated with cholesterol synthesis in empty vector or pcDNA-*Ppara* transfected *Acot12*$^{+/+}$ and *Acot12*$^{-/-}$ primary hepatocyte. All data are represented as mean ± SEM. Statistical differences among four groups were performed using one-way ANOVA, followed by Turkey's multiple comparison test. *n.s* = non-significant; **P* < 0.05; ***P* < 0.01; ****P* < 0.001; *****P* < 0.0001.

**Supplementary Figure 15.** Effect of fenofibrate treatment into *Acot12*$^{+/+}$ or *Acot12*$^{-/-}$ primary hepatocyte. **a** Expression level of *Ppara* in vehicle and fenofibrate treated *Acot12*$^{+/+}$ and *Acot12*$^{-/-}$ primary hepatocyte. Transfection efficiency confirmed by qRT-PCR. **b** Cellular acetyl CoA level in vehicle and fenofibrate treated *Acot12*$^{+/+}$ and *Acot12*$^{-/-}$ primary hepatocyte. **c** Cellular FFA (left) and TC (right) level in vehicle and fenofibrate treated *Acot12*$^{+/+}$ and *Acot12*$^{-/-}$ primary hepatocyte. **d** Representative image of BODIPY$^{493/503}$ staining in vehicle and fenofibrate treated *Acot12*$^{+/+}$ and *Acot12*$^{-/-}$ primary hepatocyte. Scale bars, 100 μm. **e**

Representative image of filipin staining in vehicle and fenofibrate treated *Acot12*$^{+/+}$ and *Acot12*$^{-/-}$ primary hepatocyte. **f** Expression level of genes associated with DNL pathway in vehicle and fenofibrate treated *Acot12*$^{+/+}$ and *Acot12*$^{-/-}$ primary hepatocyte. **g** Expression level of genes associated with cholesterol synthesis in vehicle and fenofibrate treated *Acot12*$^{+/+}$ and *Acot12*$^{-/-}$ primary hepatocyte. All data are represented as mean ± SEM. Statistical differences among four groups were performed using one-way ANOVA, followed by Turkey's multiple comparison test. *n.s* = non-significant; **$P$ < 0.01; ***$P$ < 0.001; ****$P$ < 0.0001.

**Supplementary Figure 16.** Expression level of genes involved in fatty acid oxidation which of PPARα direct target in 6 weeks vehicle or fenofibrate (FF) administered HFHCD *Acot12*$^{+/+}$ or *Acot12*$^{-/-}$ mice liver. All data are represented as mean ± SEM. Statistical differences among four groups were performed using two-way ANOVA, followed by Sidak's multiple comparison test. *n.s* = non-significant; *$P$ < 0.05; **$P$ < 0.01; ****$P$ < 0.0001.

**Supplementary Figure 17.** Pathological onset of fatty liver in *Acot12*$^{-/-}$ mice developed before increasing of adipose tissue. **a** Hematoxylin and eosin staining of liver and in HFD *Acot12*$^{+/+}$ or *Acot12*$^{-/-}$ mice. Scale bars, 100 μm. **b** The levels of hepatic TG (left) and total cholesterol (right) in HFD *Acot12*$^{+/+}$ or *Acot12*$^{-/-}$ mice. ($n$ = 3 mice per group) **c** Plasma membrane staining of iWAT in HFD *Acot12*$^{+/+}$ or *Acot12*$^{-/-}$ mice. Scale bars, 200μm. **d** Adipocyte counting (left) and area (right) per field was analyzed by Image J. All data are represented as mean ± SEM. Statistical differences between two groups were determined using multiple *t*-test with Holm-Sidak method. *n.s* = non-significant; *$P$ < 0.05; **$P$ < 0.01.

**Supplementary Figure 18.** Acot12 deficiency induces hepatic lipid accumulation independently of adipose tissue. **a** The transwell co-culture of primary adipocyte and primary hepatocytes isolated from *Acot12*$^{+/+}$ or *Acot12*$^{-/-}$ mice. Schematic diagram represented each transwell co-culture (upper panel). Representative image of BODIPY staining in co-culture of primary adipocyte and primary hepatocytes (lower). Scale bars, 200μm. **b** BODIPY-positive area in primary adipocyte (left) and primary hepatocyte (right) were measured by ImageJ and represented by bar graph. **c** Expression level of genes involved in lipid transport (left) lipogenesis (right) in *Acot12*$^{+/+}$ primary hepatocyte co-cultured with *Acot12*$^{-/-}$ primary adipocyte compared to *Acot12*$^{+/+}$ and *Acot12*$^{-/-}$ and primary hepatocyte co-cultured with *Acot12*$^{+/+}$ and *Acot12*$^{-/-}$ primary adipocyte, respectively. All data are represented as mean ± SEM. Statistical differences between two groups were determined using multiple paired *t*-test with Holm-Sidak method (**a**); Statistical differences in gene expression among three groups were performed using two-way ANOVA, followed by Tukey's multiple comparison test (**b, c**). *n.s* = non-significant; **P* < 0.05; ***P* < 0.01; ****P* < 0.001; *****P* < 0.0001.

**Supplementary figure 1**

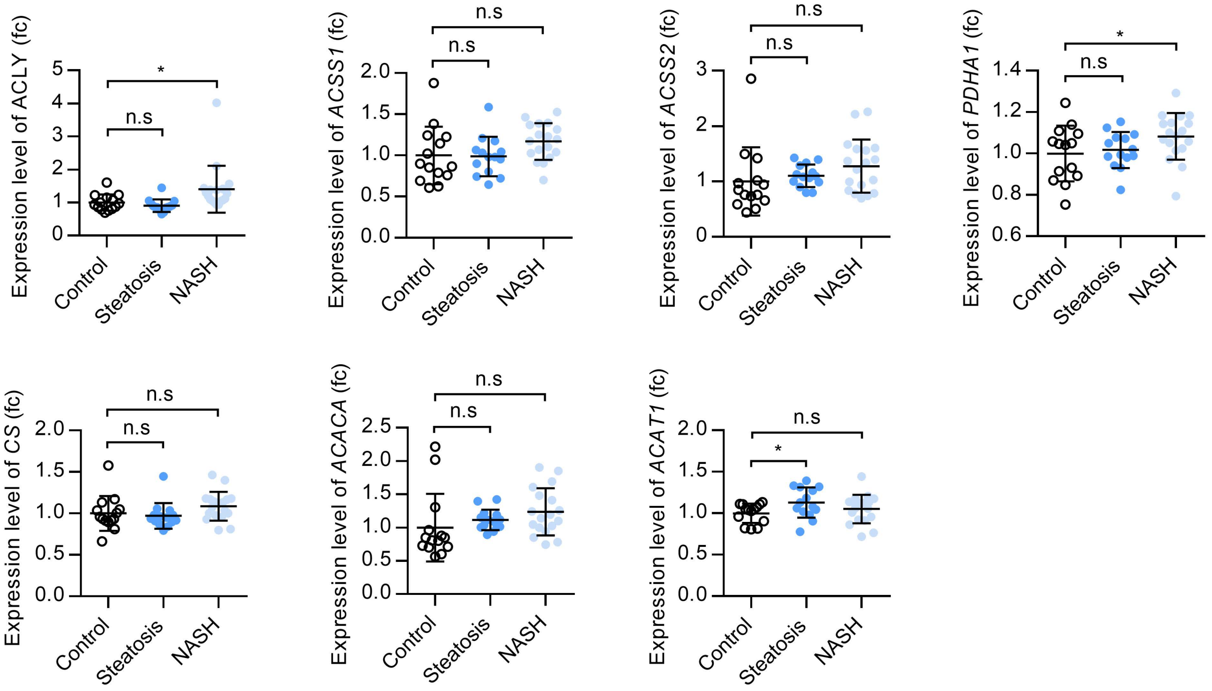

**Supplementary figure 2**

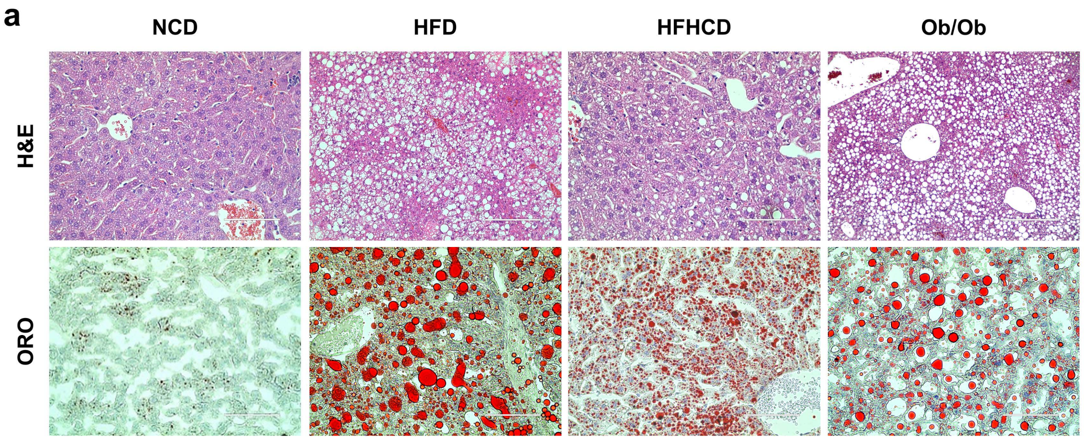


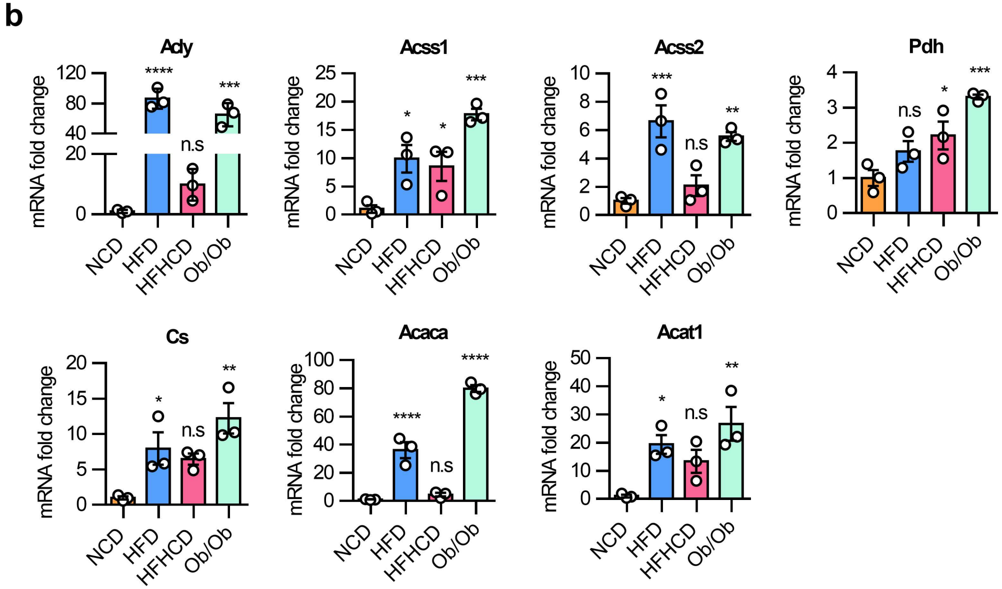

**Supplementary figure 3**

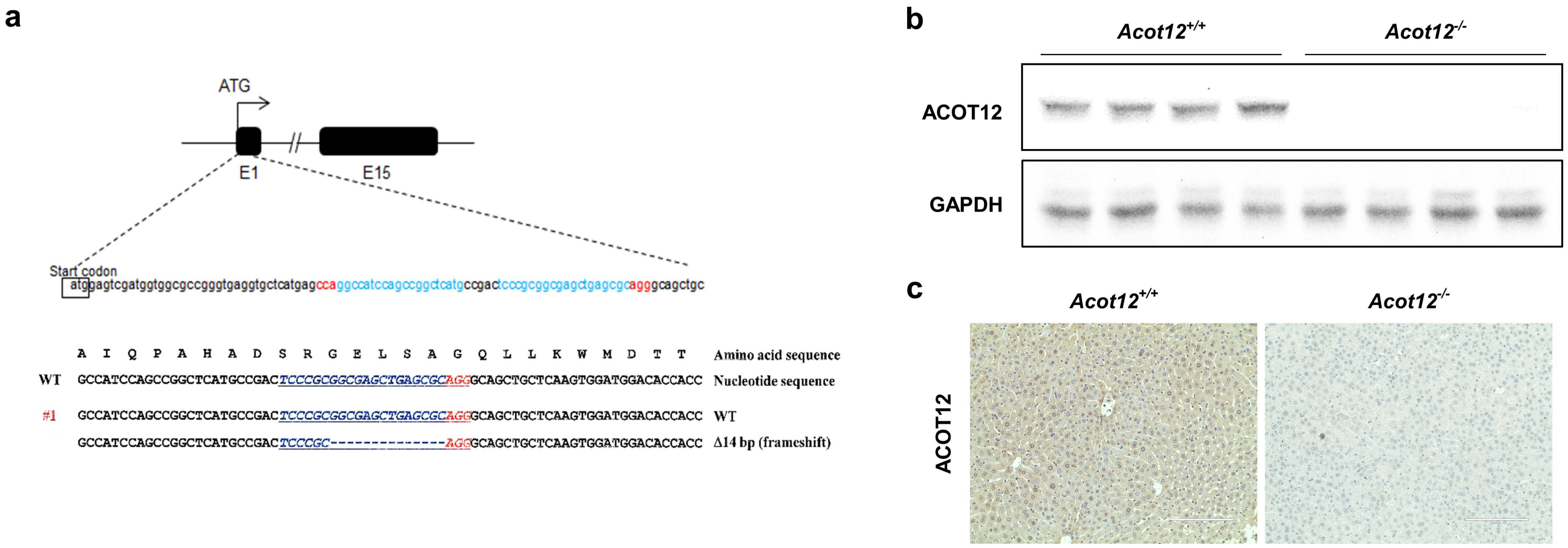

# Supplementary figure 4

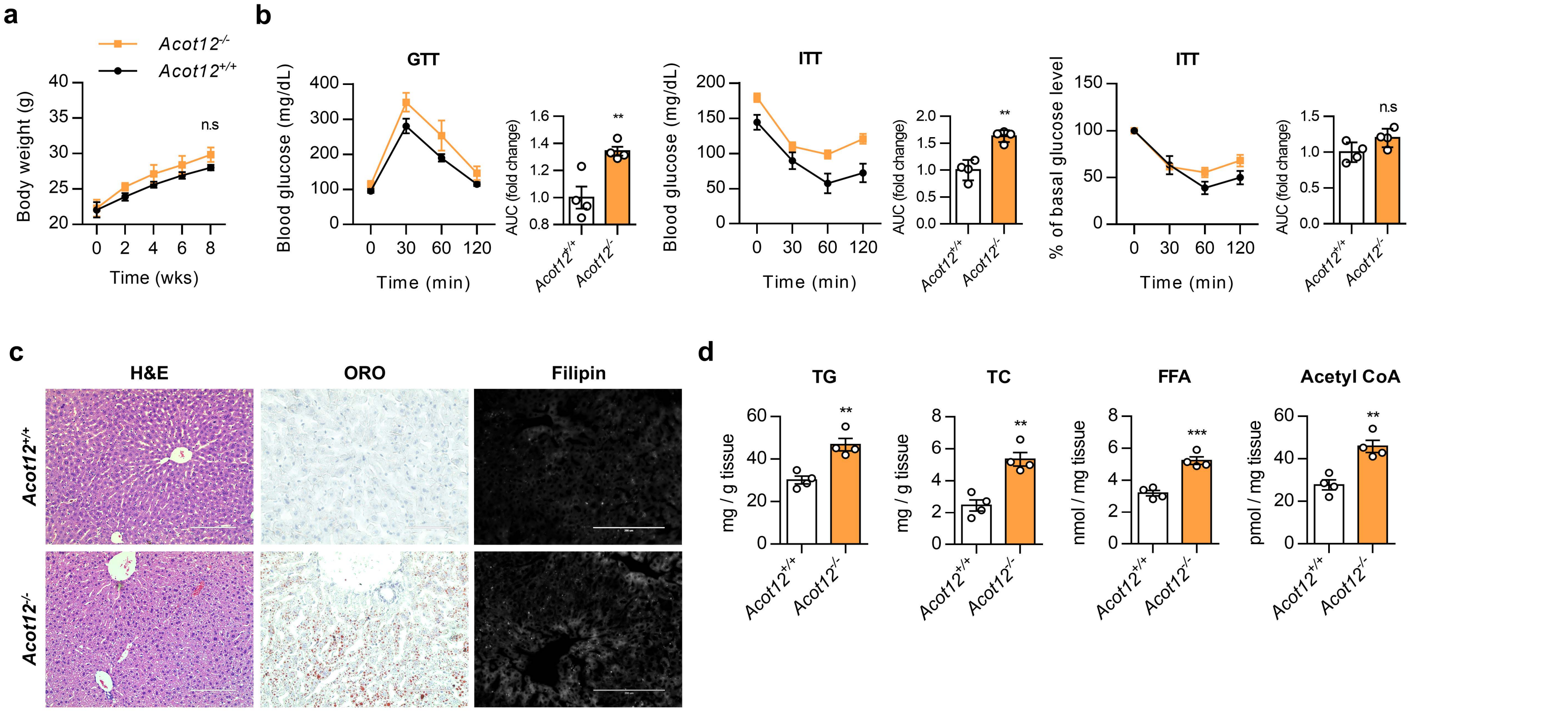

**Supplementary figure 5**

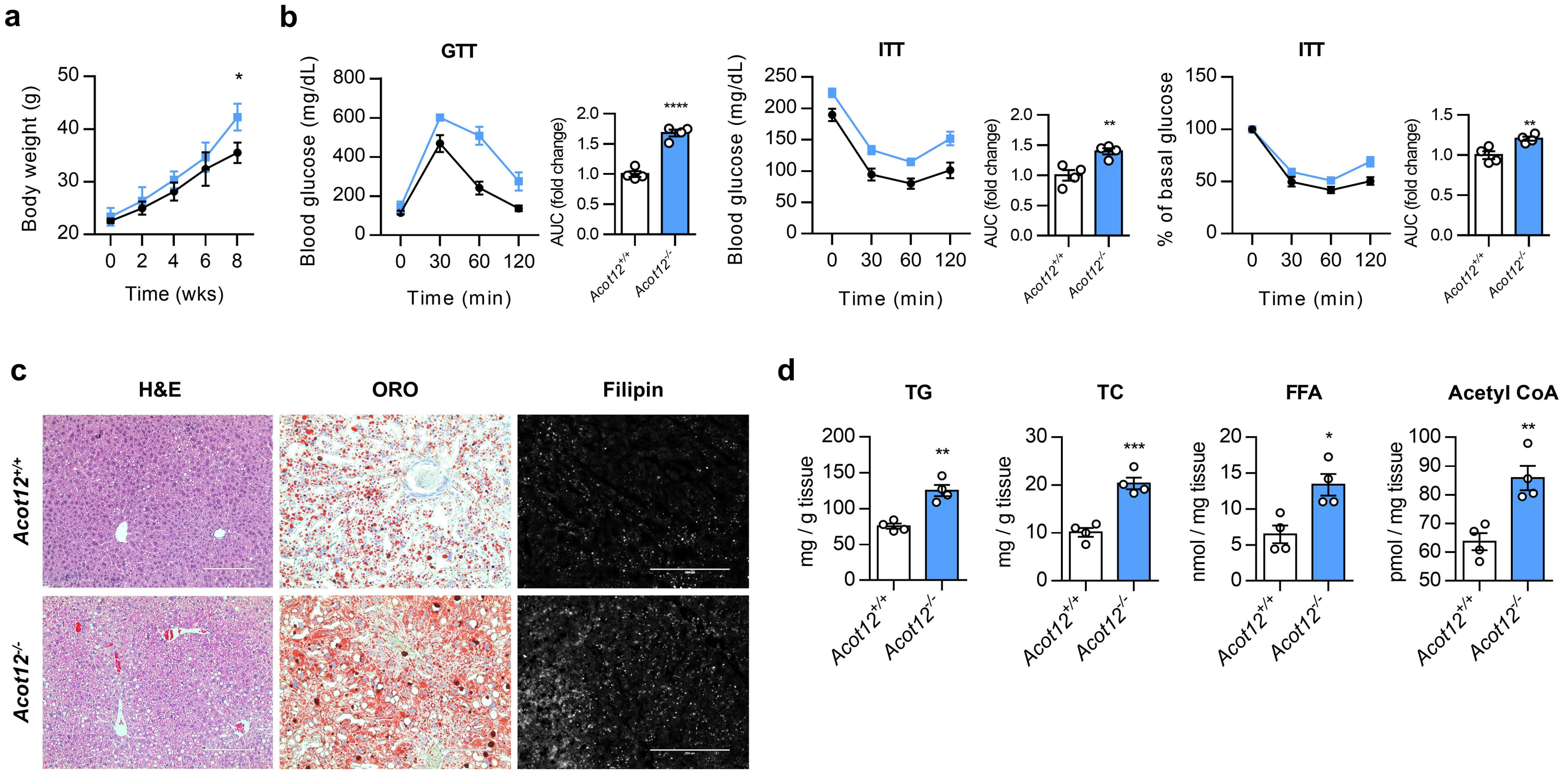

# Supplementary figure 6

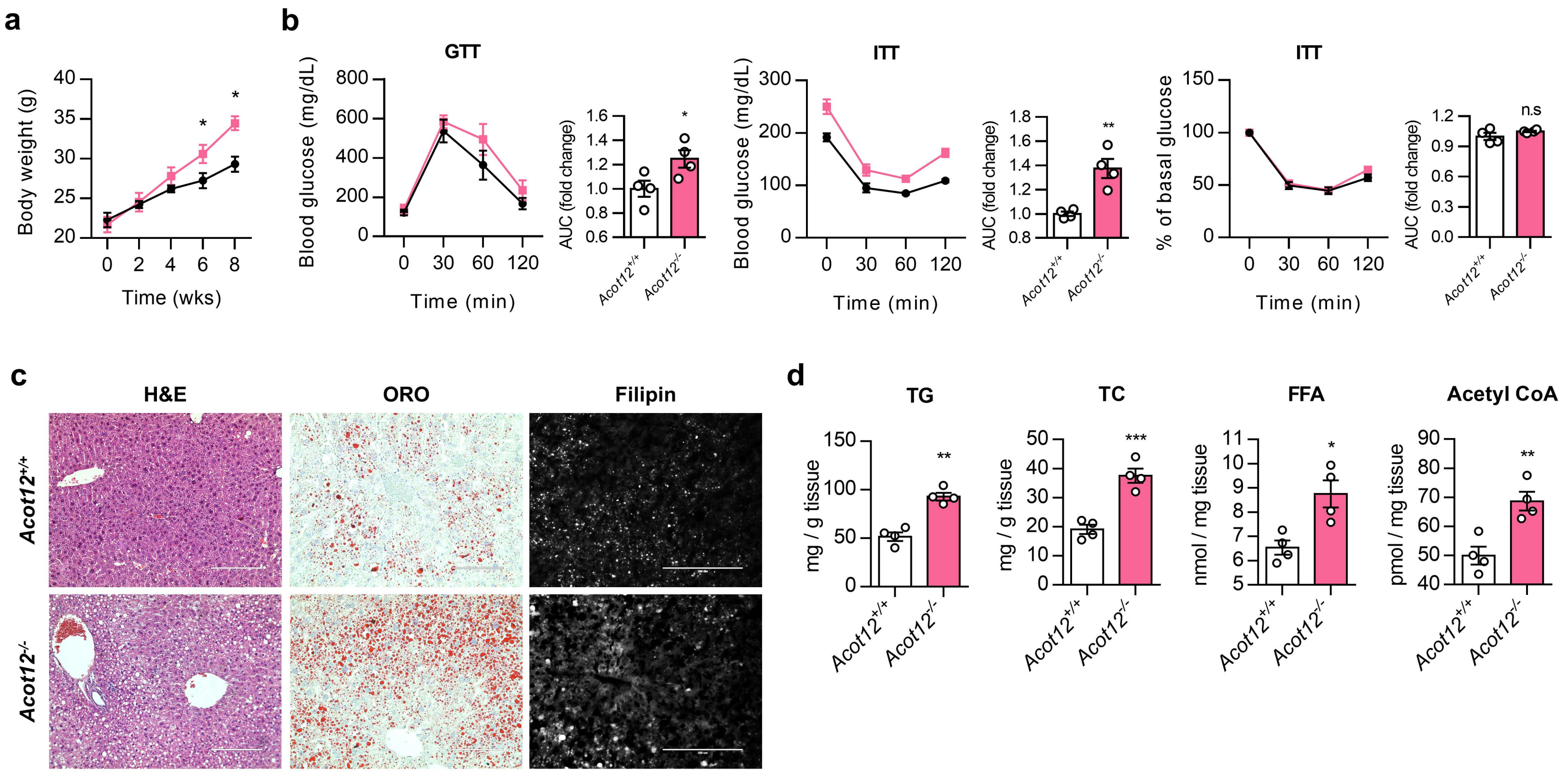

## Supplementary figure 7

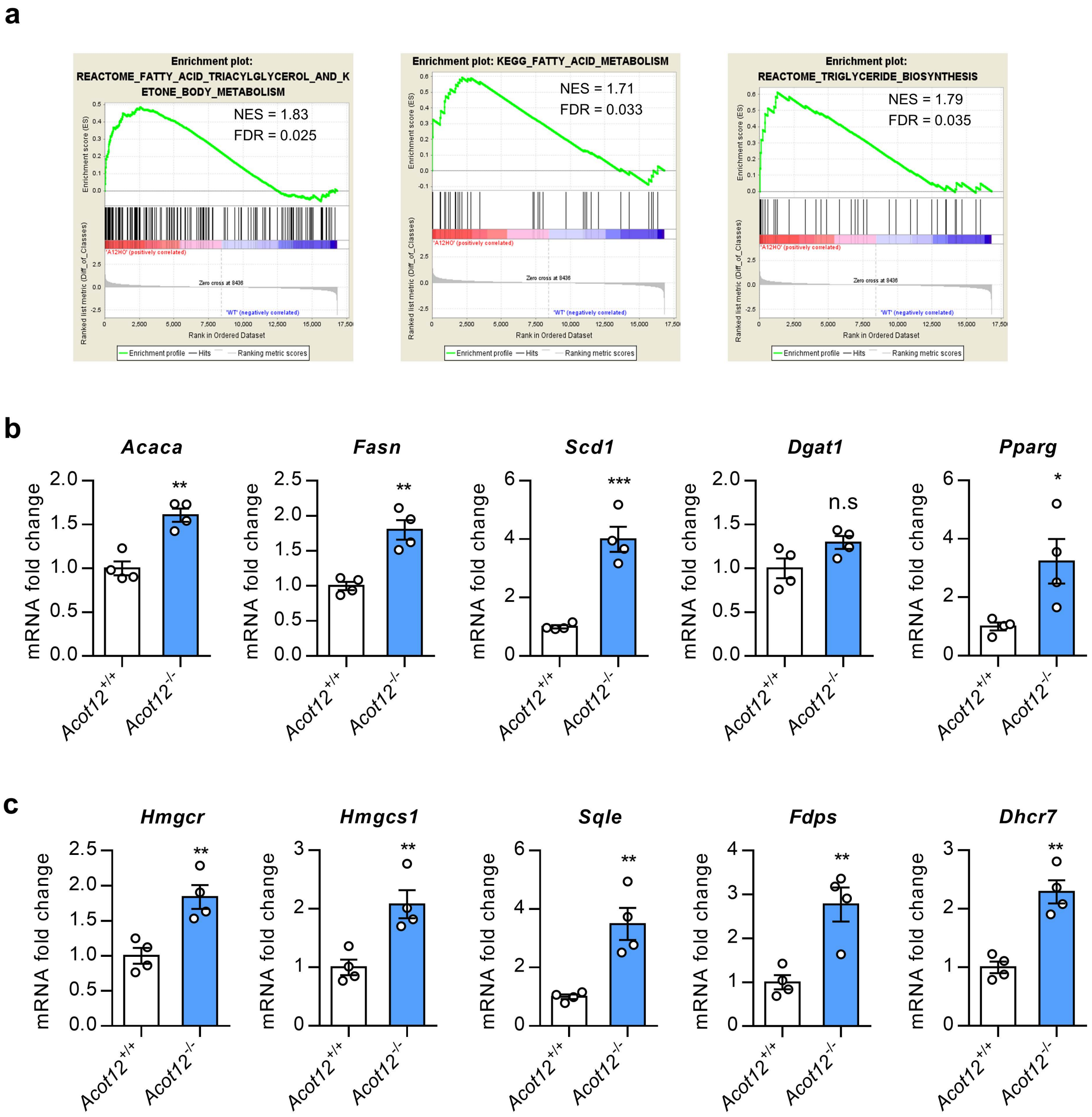

Supplementary figure 8

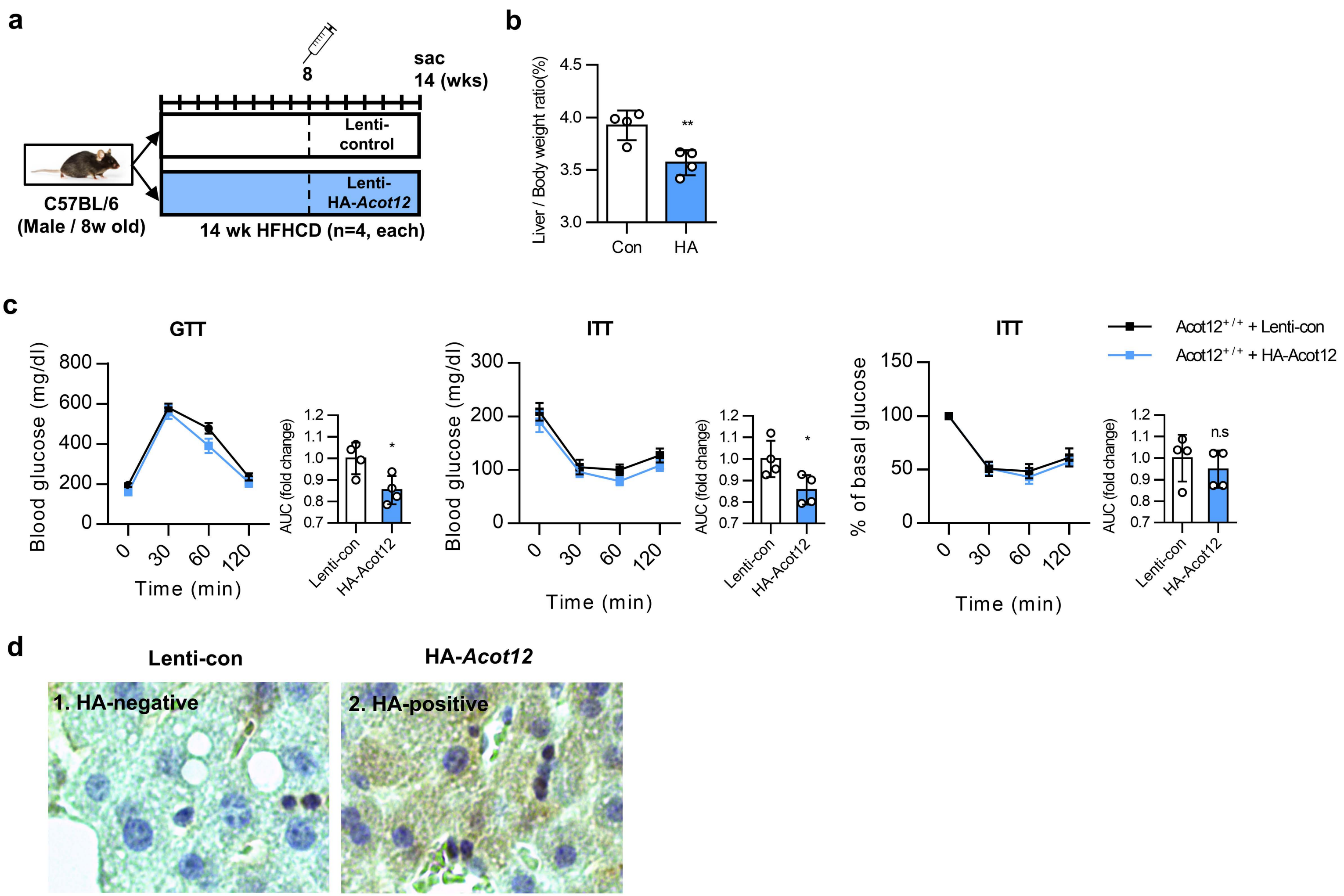

# Supplementary figure 9

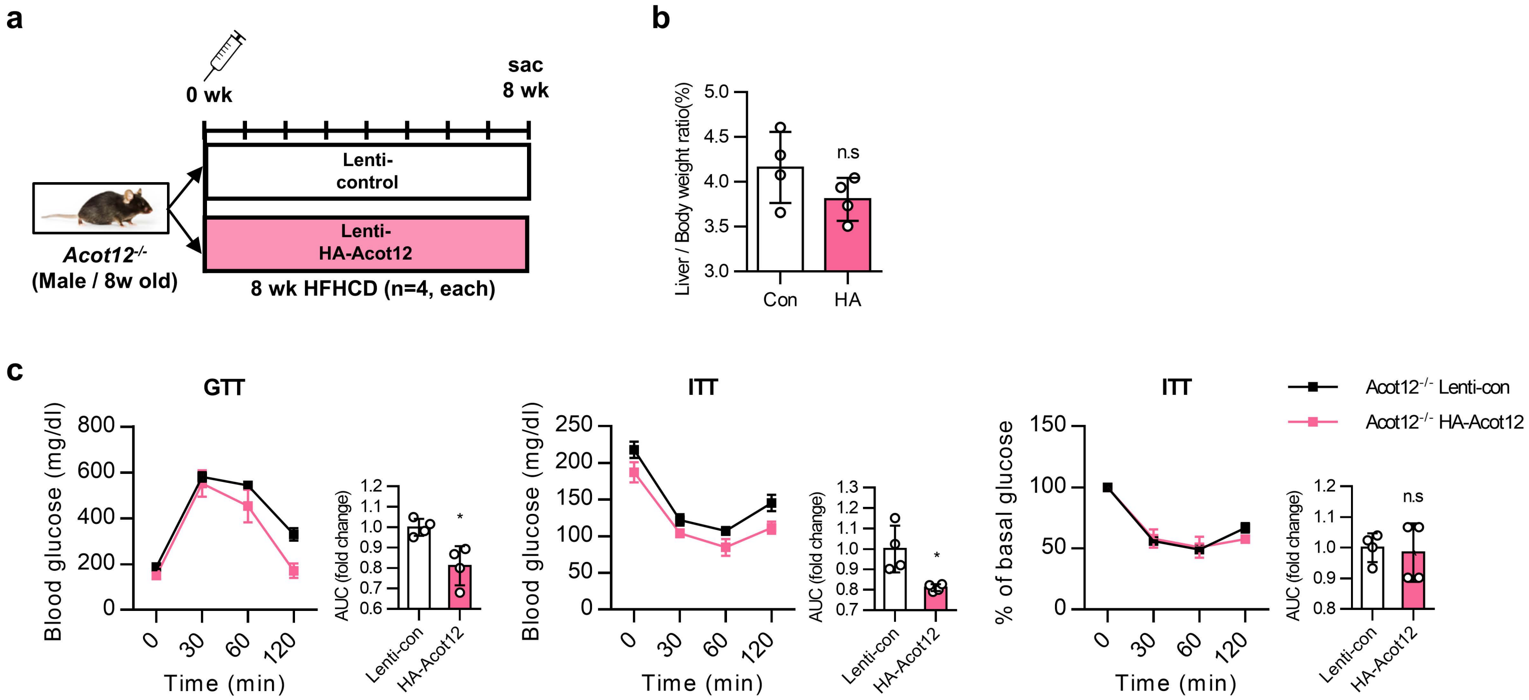

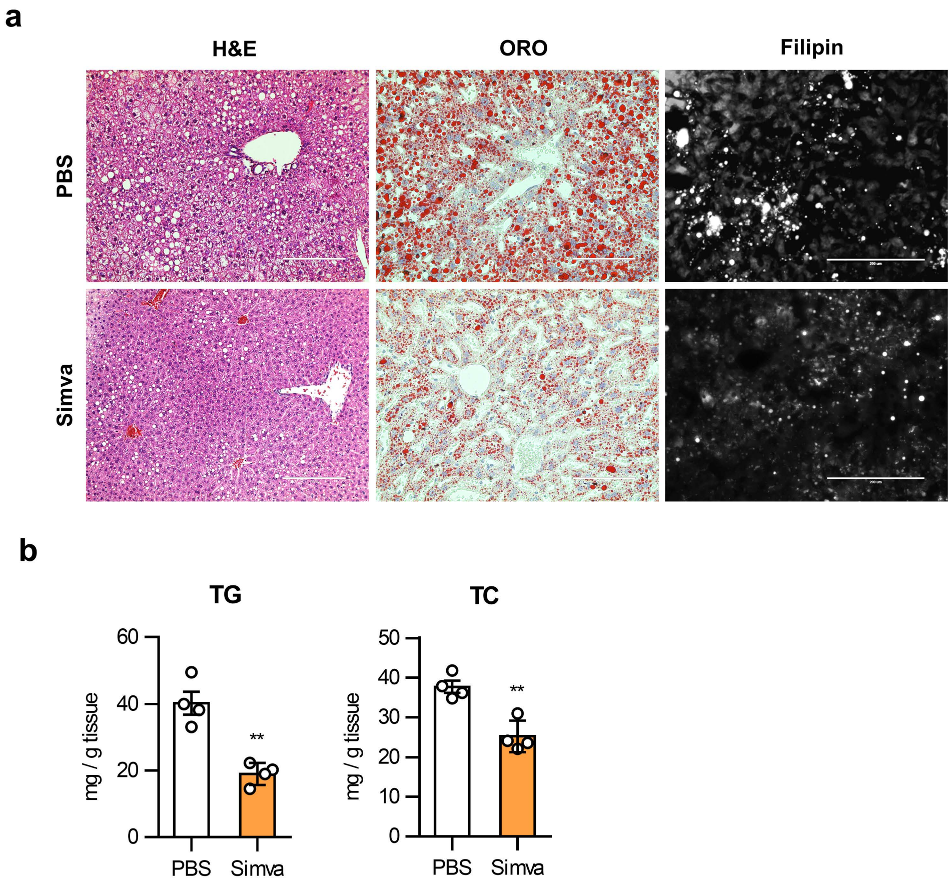
a
H&E
ORO
Filipin
PBS
Simva
b
TG
TC
mg / g tissue
60
40
20
0
50
30
10
**
PBS
Simva

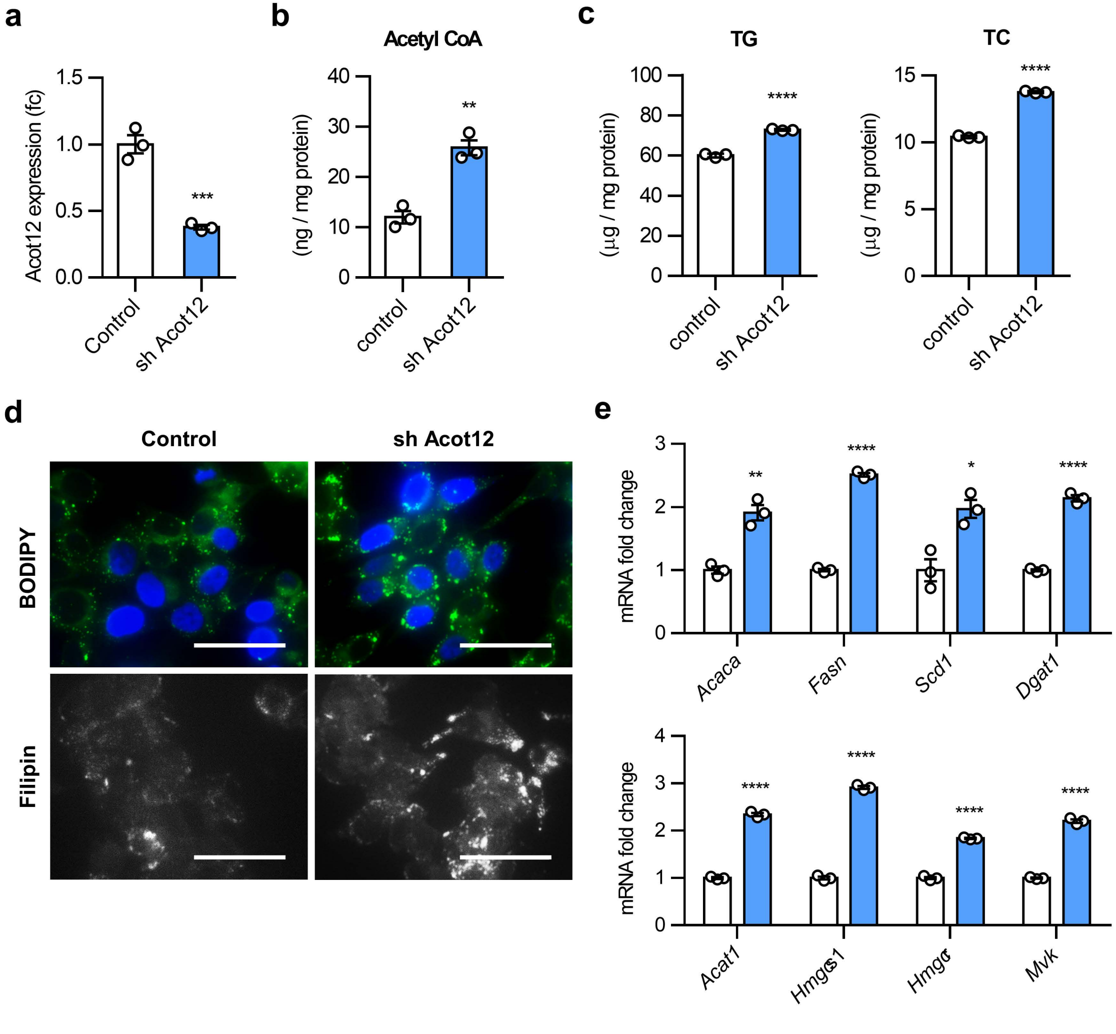
a
Acot12 expression (fc)
1.5
1.0
0.5
0.0
***
Control
sh Acot12
b
Acetyl CoA
(ng / mg protein)
40
30
20
10
0
**
control
sh Acot12
c
TG
(μg / mg protein)
100
80
60
40
20
0
****
control
sh Acot12
TC
(μg / mg protein)
15
10
5
0
****
control
sh Acot12
d
Control
sh Acot12
BODIPY
Filipin
e
mRNA fold change
3
2
1
0
**
****
*
****
Acaca
Fasn
Scd1
Dgat1
mRNA fold change
4
3
2
1
0
****
****
****
****
Acat1
Hmgcs1
Hmgcr
Mvk

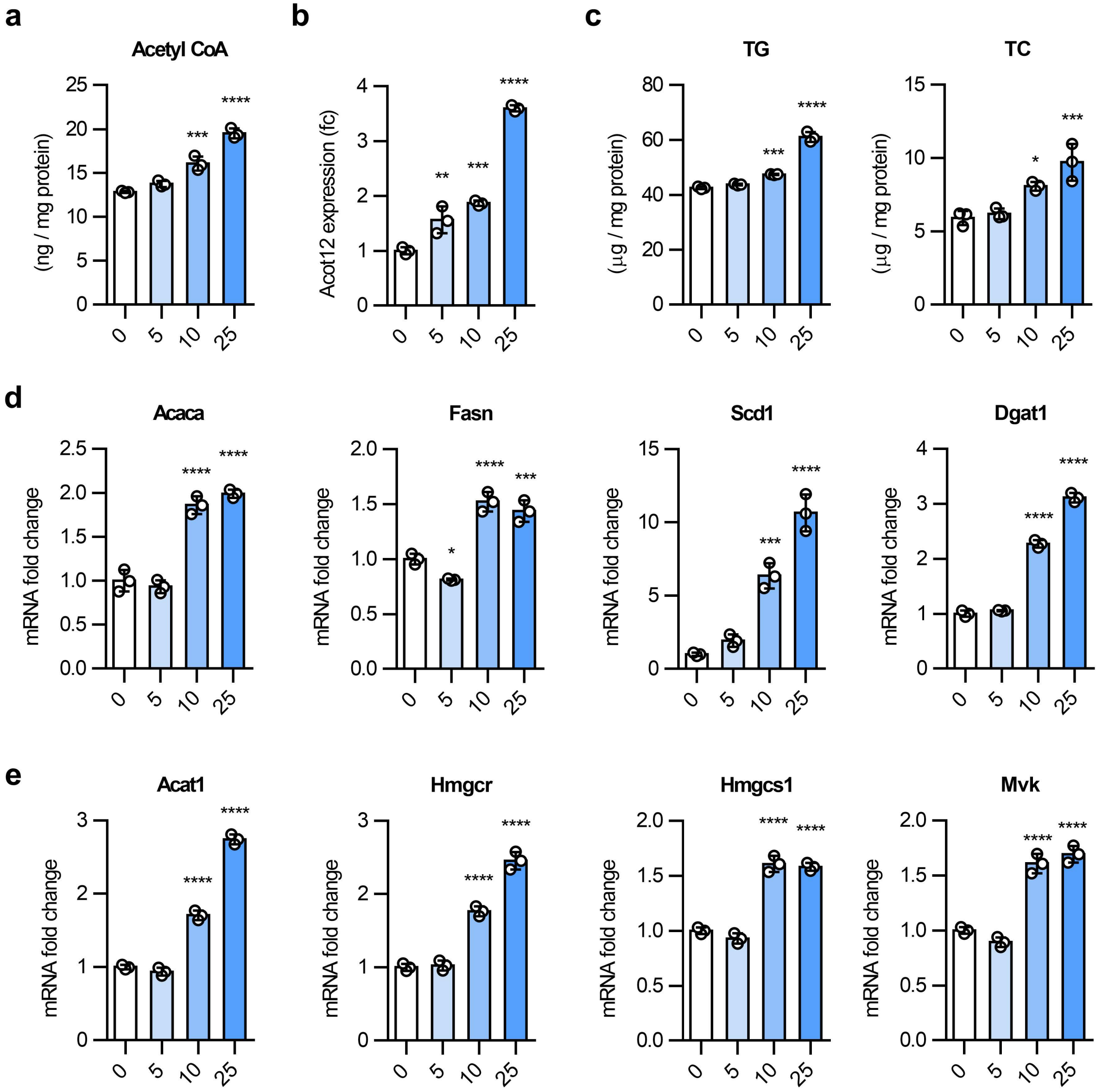
a
Acetyl CoA
(ng / mg protein)
b
Acot12 expression (fc)
c
TG
(µg / mg protein)
TC
(µg / mg protein)
d
Acaca
Fasn
Scd1
Dgat1
mRNA fold change
e
Acat1
Hmgcr
Hmgcs1
Mvk
mRNA fold change
0
5
10
25

**Supplementary figure 13**

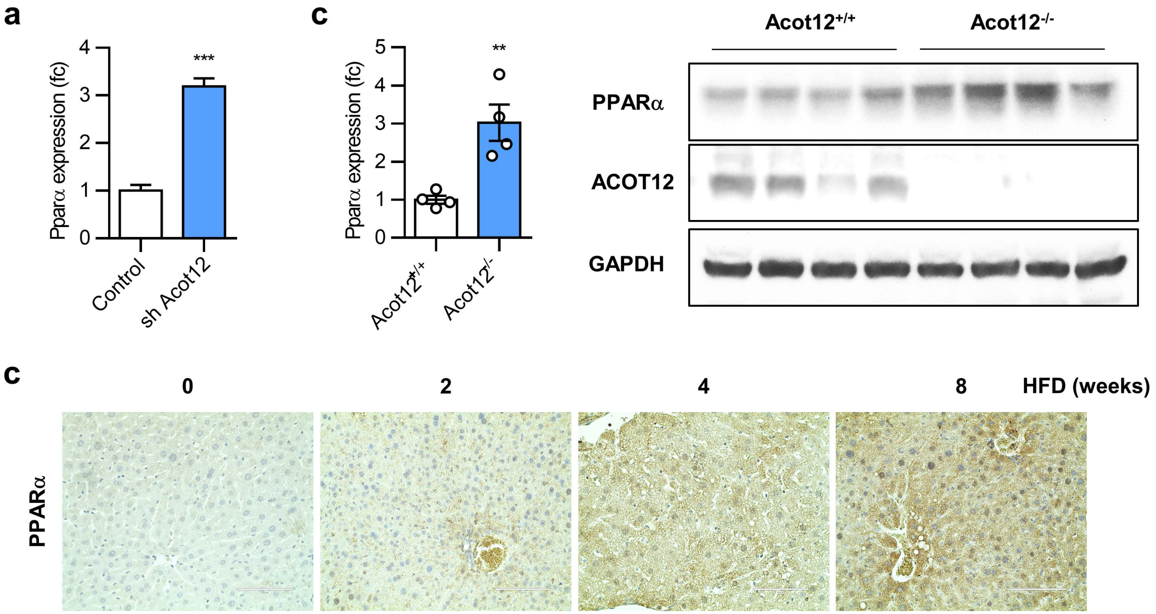

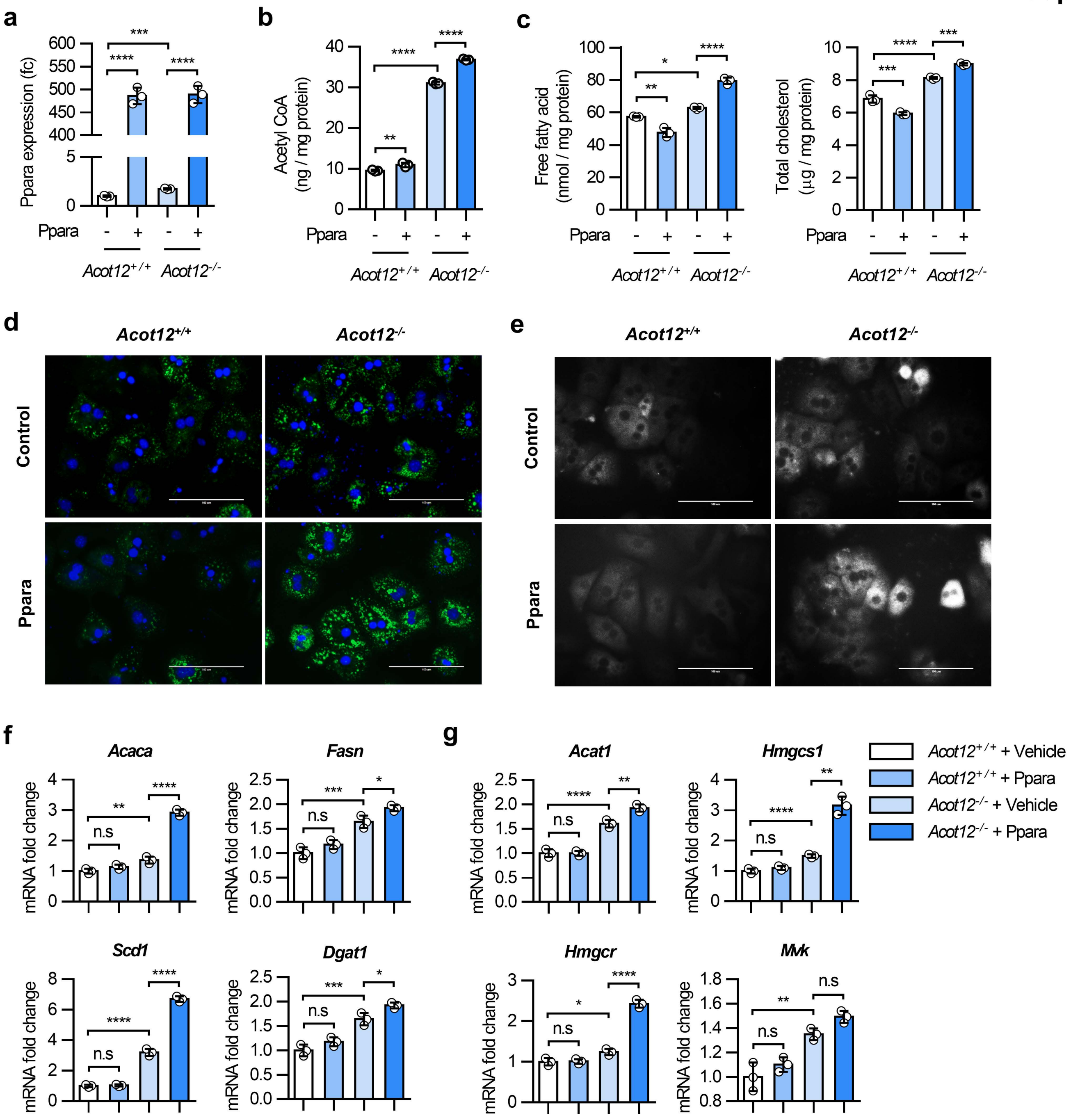
a
b
c
Ppara expression (fc)
Acetyl CoA (ng / mg protein)
Free fatty acid (nmol / mg protein)
Total cholesterol (µg / mg protein)
Ppara
Acot12+/+
Acot12-/-
d
e
Control
Ppara
f
g
Acaca
Fasn
Scd1
Dgat1
Acat1
Hmgcs1
Hmgcr
Mvk
mRNA fold change
Acot12+/+ + Vehicle
Acot12+/+ + Ppara
Acot12-/- + Vehicle
Acot12-/- + Ppara

**Supplementary figure 15**

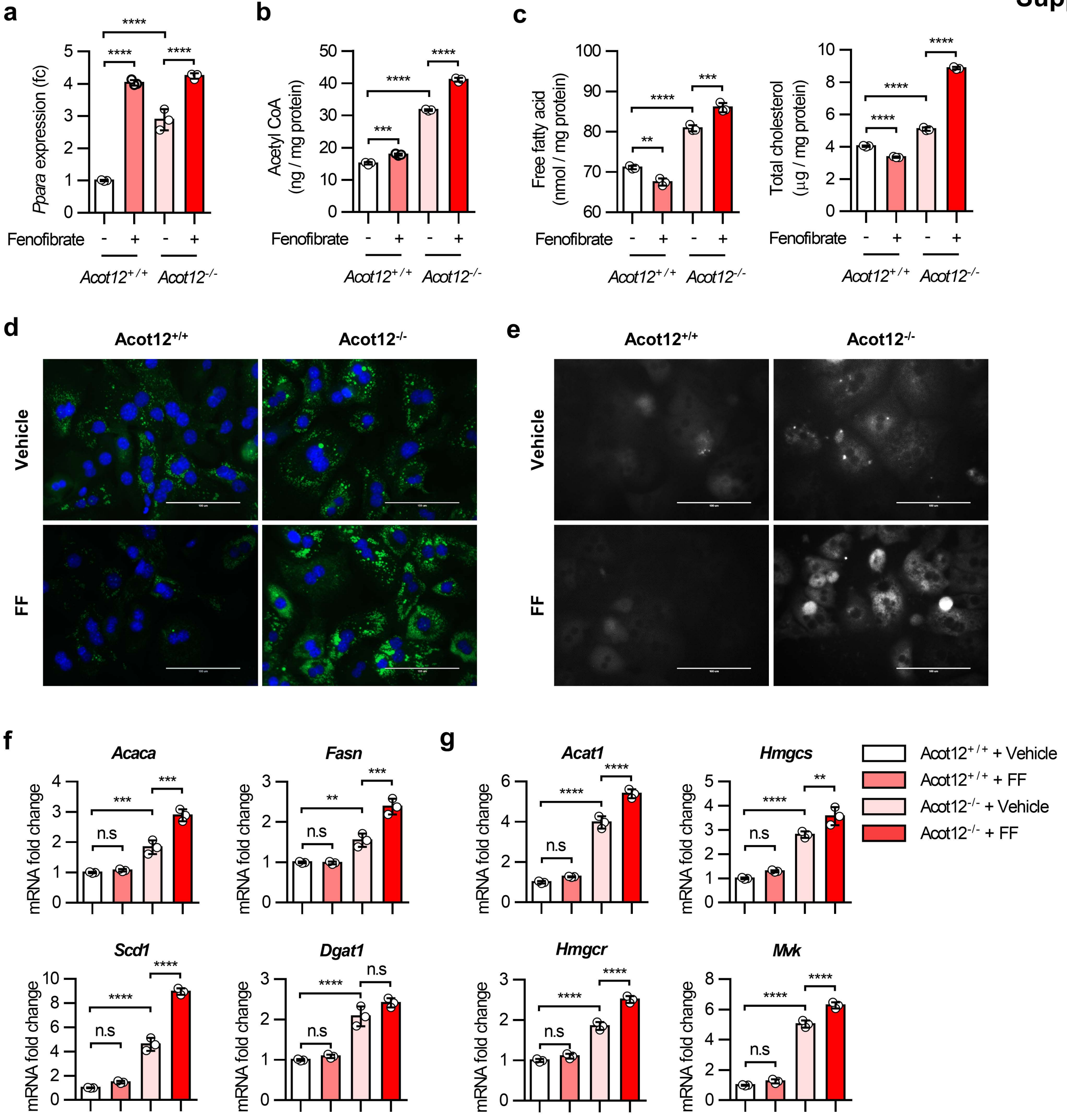

**Supplementary figure 16**

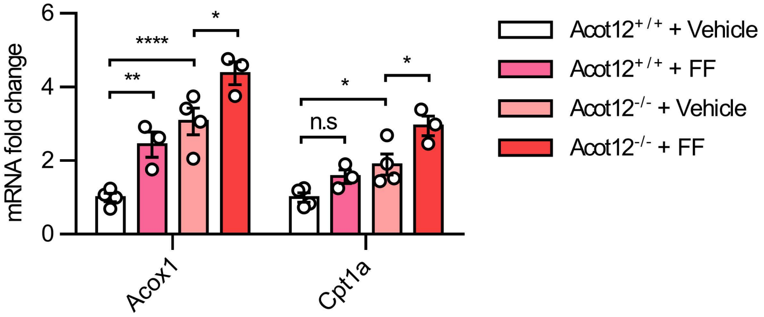

## Supplementary figure 17

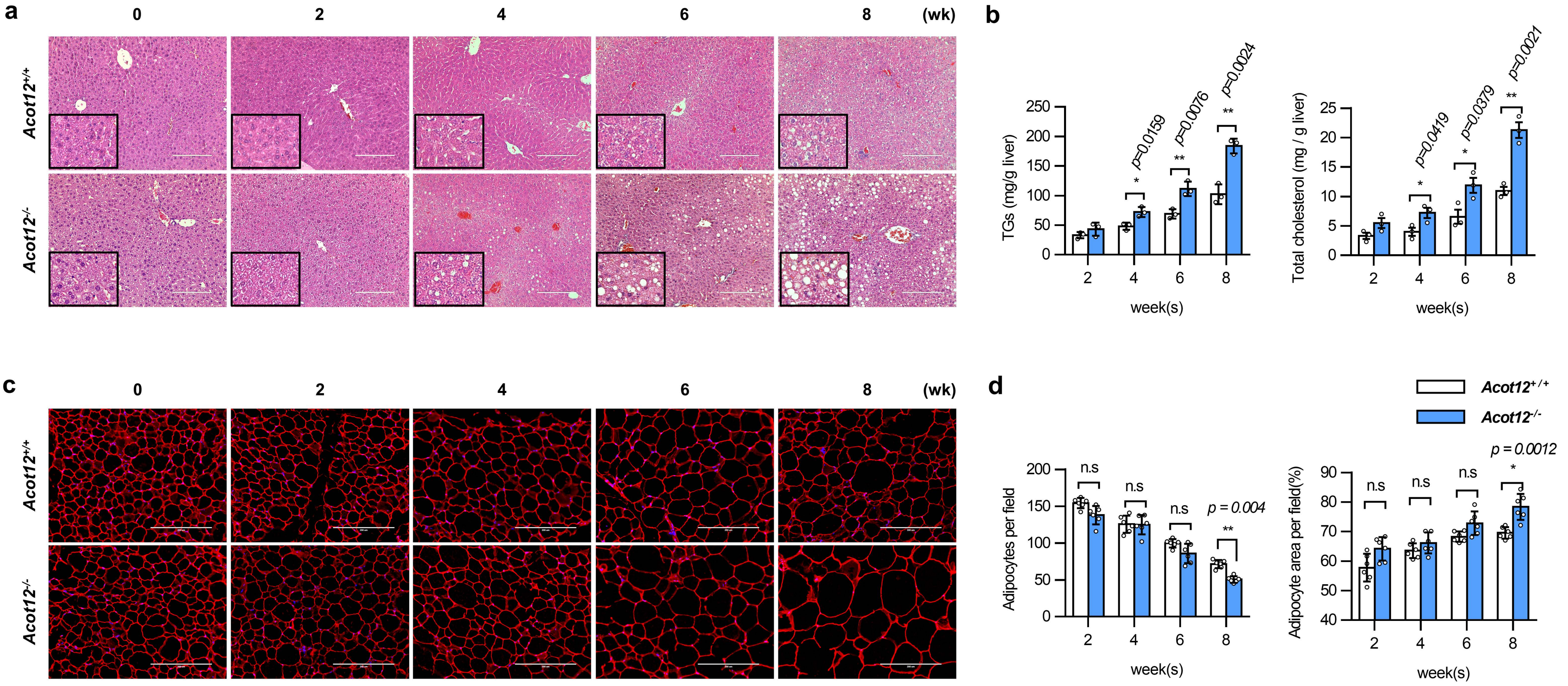

# Supplementary figure 18

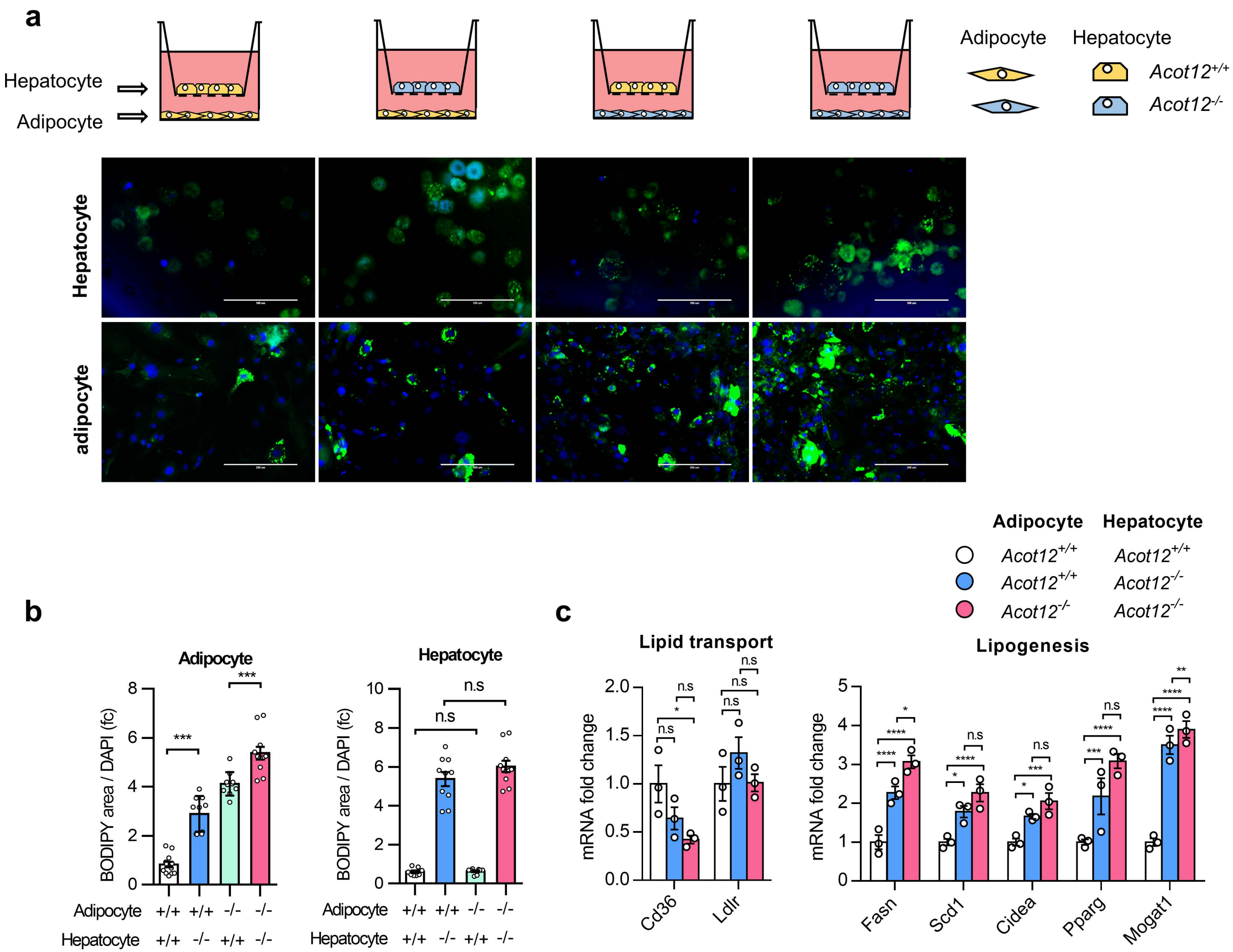

## Supplementary Table 1

| Gene symbol | Forward | Reverse |
|---|---|---|
| *Acaca* | ACATTCCGAGCAAGGGATAAG | GGGATGGCAGTAAGGTCAAA |
| *Acat1* | CATGATGGCCTCTCAAAGTCT | TGTTGCTCCTCTGCTCATTAC |
| *Acly* | CGGGAGGAAGCTGATGAATAATG | GTCAAGGTAGTGCCCAATGAA |
| *Acot12* | CCACCACCTTGGAGAAGATAAA | GTGAGGTCAGACAAGAGATGATAAG |
| *Acss1* | GAGCATGAGCAGTGAAGACA | CATGGCGGCATACAGTAGATAG |
| *Acss2* | CACCTTCTGGCAAACAGAAAC | CTACACCGAAGAATGGGAAAGA |
| *Cs* | CGAGACTACATCTGGAACACAC | CAAACTCTCGCTGACAGGAATA |
| *Pdh* | AGAGAGGATGGGCTCAAGTA | CAAGTGACAGAAACCACGAATG |
| *Fasn* | AGACCCGAACTCCAAGTTATTC | GCAGCTCCTTGTATACTTCTCC |
| *Scd1* | CAACTTCACCACGTTCTTCATC | CCCGTCTCCAGTTCTCTTAATC |
| *Cidea* | GCAACCAAAGAAATCGGGAATAG | CTCGTACATCGTGGCTTTGA |
| *Pparg* | CTGGCCTCCCTGATGAATAAAG | AGGCTCCATAAAGTCACCAAAG |
| *Mogat1* | GACCTCAGAGCAGATTGAAGAG | GTGCTCCGGAATCCCATATT |
| *Dgat1* | GGCCTTACTGGTTGAGTCTATC | GTTGACATCCCGGTAGGAATAA |
| *Cd36* | CTGGGACCATTGGTGATGAAA | CACCACTCCAATCCCAAGTAAG |
| *Ldlr* | ATCCACCGCAACATCTACTG | GGAACAGTGTCCTCCTCTTTAC |
| *Srebf1* | ACTTCCCTGGCCTATTTGACC | GGCATGGACGGGTACATCTT |
| *Hmgcr* | CTTGTGGAATGCCTTGTGATTG | AGCCGAAGCAGCACATGAT |
| *Hmgcs1* | AAATGCCAGACCTACAGGTGG | ATGCTGCATGTGTGTCCCA |
| *Mvk* | GGAGCAACTGGAGAAGCTAAA | TGCCAGGTACAGGTAGAGAA |
| *Sqle* | AGAGCCCGACAGGATAGTT | GATGGGCATTGAGACCTTCTAC |
| *Fdps* | TCGGGTGAAAGCACTGTATG | GCACTGCTCTATGAGACTCTTG |
| *Dhcr7* | CCTGACTTCTGCCACAGATT | CAGCCCATTCACCTCATACTT |
| *Ppara* | CGGTGTGTATGAAGCCATCT | TAAGGAACTCGCGTGTGATAAA |
| *Acox1* | CGCACATCTTGGATGGTAGT | GGCTTCGAGTGAGGAAGTTATAG |
| *Cpt1a* | GAAGTGTCGGCAGACCTATTT | GTCCTCCTCTCTATATCCCTGTT |
| *18s rna* | CCAGTAAGTGCGGGTCATAAG | GGCCTCACTAAACCATCCAA |
